# Emotion recognition in cross-linguistic legal context: a comparison between human-based and computational approaches

**DOI:** 10.3389/fpsyg.2026.1859697

**Published:** 2026-09-03

**Authors:** Jingyi Li, Difu Shi, Huolingxiao Kuang, Yu Weng

**Affiliations:** 1 Department of Language Science and Technology, The Hong Kong Polytechnic University, Hong Kong, Hong Kong SAR, China; 2 Institute for Computational Cosmology, Department of Physics, Durham University, Durham, United Kingdom; 3 School of Foreign Languages, Renmin University of China, Beijing, China

**Keywords:** cross-linguistic comparison, emotion recognition, legal discourse, physiological signals, sentiment analysis

## Abstract

Emotion shapes credibility assessments, judicial decision-making, and perceptions of procedural justice, yet its reliable detection in courtroom settings remains a significant methodological challenge. This study provides a multimethod evaluation of emotion-recognition approaches in cross-linguistic legal discourse using a corpus of 20 Chinese and English courtroom video clips. Eighty-eight bilingual observers provided continuous valence ratings, completed post-task Positive and Negative Affect Schedule (PANAS) assessments, and contributed electrodermal activity (EDA) and heart rate variability (HRV) recordings. Observer-perceived emotional-expression ratings were benchmarked against three analytic streams—computational, psychometric and physiological—yielding three preliminary findings. First, computational sentiment tools varied widely: cloud-based NLP services and general-purpose large language models exhibited stronger rank-order alignment with observer ratings, whereas lexicon-based and language-specific tools performed inconsistently. Second, the psychometric measure PANAS, evaluated with logistic regression, showed limited discrimination capacity. Third, among deep-learning models trained on physiological signals using Long Short-Term Memory (LSTM) architectures, the EDA-HRV fusion model yielded the highest accuracy (0.773) among several comparably performing configurations, suggesting that autonomic measures may capture affective states in courtroom interactions through the emotional contagion mechanism. Cross-linguistic analyses indicated generally stronger tool–human correspondence for Chinese clips, highlighting how linguistic and cultural factors may shape emotion detectability in legal contexts. These findings carry both methodological and practical implications for the development of specialized emotion-recognition tools in forensic and legal settings.

## 1 Introduction

Courtrooms are traditionally viewed as sites of rational argumentation. Yet emotions play a pivotal role in shaping legal narratives, participant conduct, and ultimate decisions, as emotional expressions by defendants, witnesses, judges, and jurors influence credibility assessments, decision-making, and perceptions of procedural justice (Tyler, 2006; Sunshine and Tyler, 2003; Bandes and Blumenthal, 2012). Misinterpretation or neglect of these emotional signals can lead to skewed legal judgments and erode public trust in judicial institutions (Maroney, 2006). As legal systems become increasingly mediated through digital technologies and multilingual contexts, reliable emotion recognition in legal discourse has become both a methodological and ethical imperative.

Over the past decades, research on emotion has advanced through both psychological theory and computational innovation. Emotion, understood as a multicomponent psychological and physiological response to personally significant events (Scherer, 2005; Levenson, 2011), has been modeled through discrete/categorical (e.g., Ekman, 1992; Plutchik, 1980) and dimensional frameworks (e.g., Russell, 1980; Mehrabian, 1980). Informed by these psychological frameworks, the field of affective computing (Picard, 2000) seeks to develop systems that can detect, interpret, and respond to human emotional states across multiple modalities, including facial expressions, vocal patterns, physiological signals, and textual content (Tao et al., 2007; Poria et al., 2017). Within this field, emotion recognition focuses on identifying specific emotional states, while sentiment analysis typically evaluates overall polarity or valence in texts (Munezero et al., 2014; Jiang et al., 2020). These computational approaches have enabled applications in healthcare, marketing, security, and human–computer interaction (e.g., Kamble and Sengupta, 2023; Khare et al., 2024), but their systematic application to legal contexts remains limited.

Existing methods for detecting emotion draw from both human-based and computational traditions, each with advantages and limitations. Self-report instruments, such as the Positive and Negative Affect Schedule (PANAS; Watson et al., 1988) and Russell’s circumplex model (1980), provide direct insight into subjective emotional experience but are vulnerable to response biases and emotional self-awareness constraints (Greenwald and Banaji, 1995; Althubaiti, 2016). Physiological emotion recognition methods, using signals such as electrodermal activity (EDA) and heart rate variability (HRV), offer greater objectivity because autonomic responses are difficult to consciously manipulate (Gong et al., 2024). However, their direct application to courtroom actors raises ethical and practical concerns: the direct physiological monitoring of key courtroom actors like judges, defendants, or witnesses is unfeasible in real judicial proceedings due to significant legal and logistical constraints (Szoszkiewicz and Yuste, 2025). Meanwhile, computational sentiment analysis and large language models (LLMs) have shown increasing capacity to infer affect from textual and multimodal legal data (Ash et al., 2022; Sorin et al., 2024; Han et al., 2024), yet face challenges such as detecting low-arousal or ironic expressions in formal legal language (Farias and Rosso, 2017; Šandor and Babac, 2023).

This study compares different human-based and computational approaches to emotion recognition in legal discourse in order to assess their relative strengths and limitations. On the human side, we adopt a framework that leverages the principle of emotional congruence—the alignment between the emotional states of courtroom actors and impartial third-party observers. Emotional congruence draws on well-established mechanisms of emotional contagion (Hatfield et al., 1993; Barsade, 2002), through which observers spontaneously and often unconsciously mirror or synchronize with others’ emotional expressions via facial, vocal, and physiological channels. Because such mirroring is largely automatic and pre-reflective, it is driven by the perceptible affective signals the actors emit rather than by direct access to their inner states, which renders the approach both non-intrusive and well-suited to the methodological and ethical constraints of judicial settings. Such emotional resonance can occur both in face-to-face interactions and through mediated exposure, such as watching videos of emotionally charged events (Hess and Fischer, 2013; Prochazkova and Kret, 2017). Emotional contagion can produce shared affective climates that are measurable through both self-report and physiological indicators (Michalec et al., 2025; Gashi et al., 2019), and prior research has demonstrated significant physiological synchrony between targets and observers during emotional tasks (Palumbo et al., 2017; Konvalinka and Roepstorff, 2012). Building on these findings, instead of attempting to monitor courtroom participants directly—a method that poses ethical, legal, and logistical challenges—we measure the physiological and self-reported emotional responses of observers as they watch courtroom video clips. This proxy-based approach assumes that observers’ emotional and physiological reactions, arising through affective resonance with courtroom actors, can provide an indirect yet theoretically grounded index of emotional expressions those actors display.

On the computational side, we evaluate a diverse set of sentiment analysis and emotion recognition tools, encompassing fine-tuned natural language processing (NLP) models, cloud-based enterprise application program interfaces (APIs), and LLMs. These categories represent distinct analytical paradigms: lexicon- and rule-based systems emphasize standardized affective vocabularies and heuristic scoring; cloud APIs leverage proprietary models optimized for consistency and scalability in professional applications; and LLMs draw on vast pretraining corpora and contextual inference capabilities to interpret nuanced emotional content. By systematically comparing these tools, we assess their ability to capture affective cues embedded in legal discourse, a domain characterized by formal, often emotionally muted language, culturally specific rhetorical conventions, and subtle affective signaling.

Building on these complementary human-based and computational perspectives, this study examines how reliably emotions expressed in courtroom video clips can be recognized across different modalities, integrating computational, psychological, and physiological approaches. Given the practical and ethical constraints involved in sourcing authentic judicial materials, the current study is necessarily exploratory and proof-of-concept in scale, comprising 20 clips drawn from Chinese and English court proceedings, which aims to provide preliminary evidence on the relative performance of different modalities, and identify methodological priorities for larger-scale follow-up work. Within this scope, three complementary measurement streams are systematically compared: (a) computational analyses, encompassing sentiment analysis tools and large language models; (b) psychometric self-reports, based on observers’ post-task ratings using PANAS; and (c) physiological measurements, including EDA and HRV, recorded as observers viewed the clips. To provide a common evaluative benchmark, observers also labeled the emotional valence and intensity of each video, capturing perceived affect rather than direct evidence of courtroom actors’ subjective emotions.

The analysis focuses in particular on negative emotions, which dominate courtroom interactions and exert disproportionate influence on legal outcomes. Negative affect can impair witness clarity, bias juror perceptions, and compromise judicial impartiality (Salerno and Peter-Hagene, 2015). Accurate recognition of these emotions is therefore critical for understanding their role in shaping credibility assessments, cognitive load, and moral judgments within legal decision-making. In addition, the study introduces a cross-linguistic dimension to investigate how linguistic and cultural factors shape emotional expression and its detectability. We analyze courtroom clips in both English and Chinese to examine whether the modalities correspond comparably with bilingual observers’ perceived emotion across languages. This design is motivated by the premise that affective expression is shaped by language and culture rather than universal, making cross-linguistic validation essential for the equitable application of emotion-recognition tools in multilingual legal systems.

Through this multimodal, cross-linguistic framework, the study aims to provide a structured evaluation of the relative strengths and limitations of different emotion recognition approaches. By examining how various modalities perform in legal discourse, it contributes empirical evidence that can inform the development of more context-sensitive and methodologically robust approaches to emotion recognition in judicial settings.

## 2 Materials and methods

### 2.1 Participants

The human rater participants were recruited via posters at a university in Hong Kong. They were informed that the study involved viewing video clips, wearing a non-invasive wristband for physiological recording, and completing questionnaires afterward. They would receive monetary compensation for their time.

A total of 88 participants (45 females, 43 males) aged 18–38 (*M* = 25.30, SD = 4.40) were recruited. All of them were high-level bilinguals with Chinese as their native language and English as their second language (78 participants met CEFR C1 and 10 met CEFR C2 proficiency). The sample had educational qualifications ranging from bachelor’s to doctoral levels. Individuals with legal studies backgrounds were excluded. All participants reported no history of cardiovascular or cerebrovascular disease.

### 2.2 Materials and stimuli

Authentic judicial video materials were sourced from publicly accessible platforms to ensure ecological validity and authenticity of legal discourse in both languages. Both English and Chinese materials were obtained from real-world court proceedings (English from YouTube channels *Law and Crime Network* and *Court TV*; Chinese from China Court Trial Online).^1^

An initial corpus of 20 judicial cases was systematically screened by a three-member panel. All panelists were native Chinese speakers with advanced academic training in English, enabling nuanced assessment of emotional content and legal discourse across both languages. Cases were evaluated on five criteria: (1) emotional valence and variability (negative content of varying degrees); (2) consistency of textual difficulty; (3) clip duration within a comparable range; (4) coverage of a range of legal topics (e.g., homicide, domestic violence, smuggling, intellectual property, traffic, firearms); and (5) availability of natural, unscripted courtroom discourse. This process yielded a final dataset of 10 cases (5 English, 5 Chinese), each paired with two video clips, for a total of 20 clips matched on textual difficulty. The selected clips have an average duration of 61.45 seconds (SD = 10.49). Textual difficulty of the materials was controlled to be comparable (see Supplementary Appendix A for details), and the English videos were subtitled to facilitate comprehension.

### 2.3 Procedures of the annotation experiment

This experiment was approved by the Ethics Committee of The Hong Kong Polytechnic University (Sep. 13, 2024; No. HSEARS20240912011). All participants were provided informed consent for data collection before the experiment. Publicly available court materials were used exclusively, ensuring no privacy violations.

The participants were reminded not to drink alcohol, smoke or consume foods with high caffeine within 24 h before the experiment and not to engage in vigorous exercise within 2 h before the experiment starts to ensure physiological data validity.

The experiment was conducted in a controlled, silent environment with consistent and adequate lighting, maintaining a stable indoor temperature of 25°C. Before the experimental tasks started, participants were instructed to rest for 5 min for baseline physiological measurements using an EmbracePlus wristband. During the video-watching sessions, participants’ physiological responses were continuously recorded using the same wristband. The EmbracePlus wristband is a research-grade medical wearable device that continuously monitors multiple physiological signals including HRV, EDA, skin temperature, and motion. The device has demonstrated clinically acceptable accuracy (Gerboni et al., 2023).

The experimental task involved viewing the 20 selected video clips, with the viewing order counterbalanced. Prior to each video-watching session, the experimenter provided a brief and objective introduction to the case and informed participants about the role of the speaker. During video viewing, participants were instructed to refrain from speaking or moving and were required to remain seated in a stable position throughout.

Following each video-watching session, participants completed a comprehension question, followed by the PANAS items (Chinese version, Huang et al., 2003) to measure their own state emotion. They then rated the emotion valence and intensity of the video content (i.e., “How do you perceive and rate the emotions displayed in the video?”), which reflects human judges’ perception of emotions contained in the videos. The rating scale is a 21-point Likert scale with anchor points at 0 (strongly negative), 10 (neutral), and 20 (strongly positive), to allow for the differentiation of subtle emotional variations. All the items are administered in participants’ native language, Chinese, to ensure construct validity.

### 2.4 Sentiment analysis tools

Eleven sentiment analysis tools were systematically evaluated, comprising two large language models (GPT-4o, DeepSeek-R1), two fine-tuned models (BERT-GO-Emotions, BERT-Multilingual), three NLP tools (VADER, TextBlob, HanLP), and four cloud-based NLP APIs (Google Cloud Natural Language API, Amazon Comprehend, Amazon Rekognition, Microsoft Azure Video Index). Each category offers a distinct approach to recognizing emotion in the stimuli, from the generative capabilities of LLMs to the specialized sentiment detection of fine-tuned models and the scalable solutions of cloud services. Table 1 details each tool.

**TABLE 1:** Summary of the sentiment analysis tools.

Category	Subcategory	Tool/service	Languages analyzed	Modalities	Sentiment/Emotion capability	Remarks
LLMs	General-purpose	GPT-4o	English; Chinese	Text	Zero-shot sentiment/valence with confidence	Prompting with domain constraints; identical prompts across runs; adopted the mean score of three runs
LLMs	General-purpose	DeepSeek-R1	English; Chinese	Text	Zero-shot sentiment/valence with confidence	Same as GPT-4o
Fine-tuned Transformers	Emotion classification	BERT GO-Emotions	English; Chinese (via translation)	Text	Multi-label emotions mapped to valence	Checkpoint: SchuylerH/bert-multilingual-go-emotions; document mapping to Negative/Neutral/Positive
Fine-tuned Transformers	Multilingual classification	BERT Multilingual	English (applied subset)	Text	Sentiment/emotion classification	Checkpoint: nlptown/bert-base-multilingual-uncased-sentiment; model supports many languages; study applied to English only; training corpus: Amazon, Yelp, and IMDb English review datasets
Classical/Rule-based NLP	Lexicon-based	VADER	English	Text	Polarity and intensity scores	Tuned for social media
Classical/Rule-based NLP	Lexicon-based	TextBlob	English	Text	Polarity and subjectivity	Pattern/lexicon-based
Classical/Rule-based NLP	Chinese NLP toolkit	HanLP	Chinese	Text	Polarity and intensity scores	Sentiment tokenization; model supports many languages; study applied to Chinese only
Cloud APIs	NLP	Google Cloud Natural Language API	English; Chinese	Text	Document/sentence sentiment	Language autodetect used; model supports many languages; API version: Google-cloud-language-v1 and v2; API region: Global endpoint
Cloud APIs	NLP	Amazon Comprehend	English; Chinese	Text	Sentiment and key phrases	Model supports Chinese with reduced accuracy compared to English; API region: US East—North Virginia
Cloud APIs	Vision	Amazon Rekognition	–	Visual	Face attributes/emotions	Emotion labels mapped to valence; document mapping to Negative/Neutral/Positive
Cloud APIs	Multimodal indexing	Microsoft Azure Video Indexer	English; Chinese	Text and Visual	Transcript sentiment; speaker/emotion cues	Settings: languages select (English and Mandarin); API region: East US

LLMs, language models; NLP, natural language processing; API, application program interface; BERT, bidirectional encoder representations from transformers; VADER, Valence Aware Dictionary and sEntiment Reasoner; IMDb, Internet Movie Database.

A zero-shot prompting approach (Kojima et al., 2022) with explicit task instructions and domain-specific constraints (Liu et al., 2023) was employed to elicit sentiment judgments from LLMs. The primary prompt embedded the case text and instructed the model to assess emotional valence—classifying each instance as Negative, Neutral, or Positive—and to report confidence scores. The prompt emphasized that analysis should target the speaker’s expressed emotions rather than normative judgments about legal behavior, criminal acts, or case outcomes, requiring a clear separation between factual legal content and the affective tone of the speaker’s statements, consistent with instructional prompting practices (Ouyang et al., 2022). Follow-up prompts solicited finer-grained assessments of valence intensity and an overall bias estimate. To mitigate output variability and enhance reliability (Zhu et al., 2024; Rokach, 2010), each text was evaluated three times with identical prompts, and mean scores were computed across runs. Full prompting procedures, parameter settings, and preprocessing steps are provided in Supplementary Appendix B.

### 2.5 Data processing

#### 2.5.1 Human annotation data processing

Given the nature of the selected legal discourse, the human ratings were predominantly concentrated between the scale anchors of 0 (“strongly negative”) and 10 (“neutral”) (see section 3.1), reflecting the primarily negative valence of the selected legal stimuli. To facilitate the analysis of this negative-to-neutral spectrum, the continuous 21-point scale was discretized into five specific intervals: [0–2) (Strongly negative), [2–4) (Very negative), [4–6) (Moderately negative), [6–8) (Slightly negative), and [8–10) (Neutral with slight negativity). A final interval of [10+) was designated for any positive ratings. The use of half-open intervals ensures that every possible rating is assigned to exactly one category. This categorized data served as the observer-perceived emotional-expression benchmark for evaluating the computational, psychological and physiological models’ performance.

#### 2.5.2 The conversion system of sentiment analysis outputs

To ensure comparability between computational and human ratings, outputs from all sentiment analysis tools were standardized and converted to a 21-point rating scale (0–20), consistent with the human rating scheme. Three conversion methods were applied, depending on the type of output produced by each tool.

The first method addressed numerical interval outputs generated by tools such as VADER, TextBlob, HanLP, and Google Cloud Natural Language API. These tools produce discrete scores within the range [−1, 1], where 0 represents neutral sentiment. Scores were linearly rescaled to the [0, 20] range using a simple mapping in which the midpoint (10) corresponds to neutral sentiment, preserving the relative distance between negative and positive values.

**FIGURE 1:**
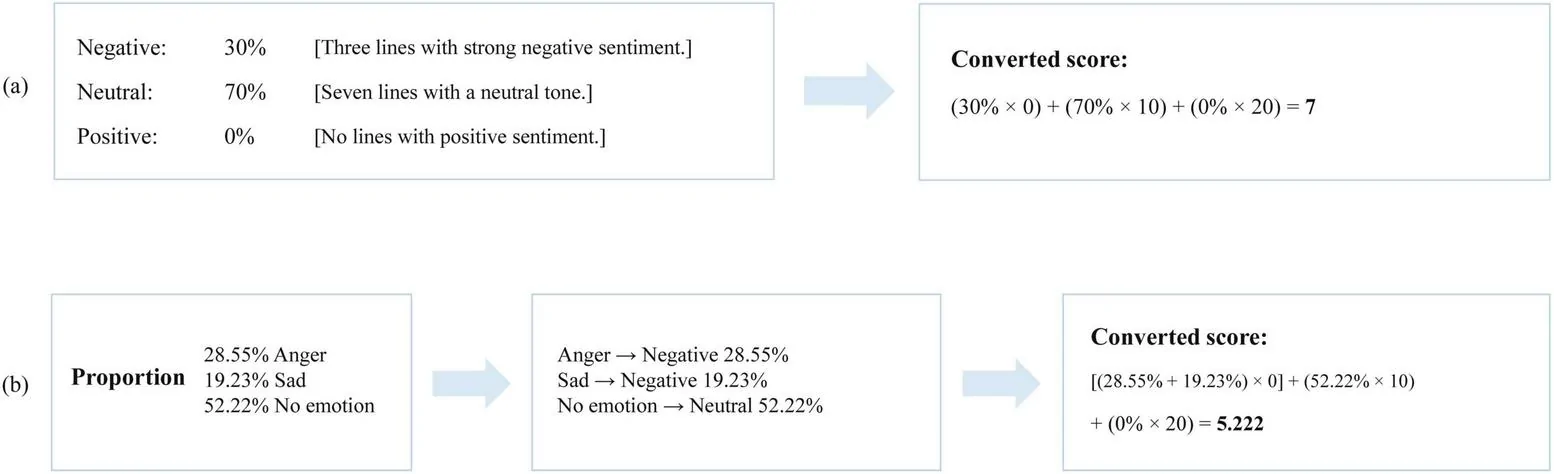
Converting examples for **(a)** probability distribution outputs and **(b)** emotional vocabulary outputs.

The second method handled probability distribution outputs generated by tools including GPT-4o, DeepSeek-R1, Amazon Comprehend, and BERT-Multilingual. For each instance, the final sentiment score was computed as the expected value of the distribution over negative, neutral, and positive classes:


Score=(P×n⁢e⁢g0)+(P×n⁢e⁢u10)+(P×p⁢o⁢s20),

where P_*neg*_, P_*neu*_, P_*pos*_ denote the predicted probabilities for each sentiment class. The weights (0, 10, 20) represent equidistant points on the human scale, ensuring a linear transformation that preserves sentiment intensity ordering (Pang and Lee, 2008). This method converts the probabilistic outputs into a continuous numerical score within the human rating range. An example is shown in Figure 1a.

The third method processed categorical emotion label outputs from tools such as BERT-GO-Emotions, Azure AI Video Indexer, and Amazon Rekognition. These tools output specific emotion words (e.g., *angry*, *sad*, *happy*), which were first mapped to sentiment categories (negative, neutral, positive) using established emotion lexicons (Mohammad and Turney, 2010). Each mapped category was then assigned an intensity weight based on its valence strength (Warriner et al., 2013). The resulting categorical probabilities were subsequently transformed into the 21-point scale using the same weighted expectation formula as in the second method (see Figure 1b).

It should be emphasized that mapping heterogeneous tool outputs (probability distributions, bounded lexicon scores, and categorical labels) onto a single 21-point scale is a standardization procedure. The anchor weights and lexicon mappings involve analyst-defined choices (researcher degrees of freedom); the resulting 21-point scores therefore index the relative ordering of sentiment across tools and are not intended to equate how each tool operationalizes emotion.

#### 2.5.3 PANAS data processing and analysis

The PANAS consists of 20 items—10 assessing positive affect and 10 assessing negative affect (Watson et al., 1988)—which allows for multiple ways of representing participants’ emotional states as model features. To examine how different representations influence model performance, we systematically compared a set of alternative feature configurations, including (1) the two positive and negative total scores (2 features), (2) the 20 individual items (20 features), (3) the 10 positive items (10 features), and (4) the 10 negative items (10 features). This range of configurations allowed us to compare coarse-grained versus fine-grained emotional representations and to assess whether separating positive and negative subscales offered any modeling advantages.

Each observer’s PANAS response after each clip was treated as one participant-clip observation, yielding 1,760 observations from 88 observers and 20 clips. We implemented class-balanced L2-regularized logistic regression using scikit-learn 1.6.1 with NumPy 2.1.3 in Python 3.10.15 to examine whether PANAS self-reports aligned with the observer-perceived emotional-expression labels. To avoid participant-level leakage, performance was estimated using participant-level 5-fold cross-validation, so that observations from the same participant never appeared in both training and testing folds. Hyperparameters were tuned with nested cross-validation (an inner selection loop within the outer folds), and we evaluated dimensionality reduction (PCA and univariate feature selection) as robustness checks (Supplementary Appendix C).

#### 2.5.4 Physiological signal processing

Physiological recordings provided the time-series inputs for subsequent deep learning analyses, comprising continuous EDA and HRV signals collected during stimulus presentation. EDA signals were sampled at 4 Hz and preprocessed in two stages. In the first stage, we computed basic descriptive statistics for each recording, including the mean, standard deviation, maximum, and minimum values of the raw EDA signal. In the second stage, we extracted indices of skin conductance response (SCR) amplitude and their corresponding descriptive statistics (mean, standard deviation, maximum, minimum, and count). SCR amplitudes were derived using Ledalab (version 3.4.9),^2^ a MATLAB-based toolbox for EDA analysis. The signal was first processed with a first-order low-pass Butterworth filter with a 5 Hz cutoff to reduce high-frequency noise. Continuous Decomposition Analysis (CDA) (Benedek and Kaernbach, 2010) was then applied to optimize the extraction of phasic SCR components. The basic descriptive statistics of the raw EDA signal and SCR amplitudes were combined into pre-task engineered summary features, which served as scalar inputs for modeling.

HRV was derived from blood volume pulse (BVP) signals sampled at 64 Hz. BVP peak detection combined multiple algorithms to ensure robust identification across varying signal qualities; RR intervals were calculated and physiologically validated by excluding intervals outside the 300–2,000 ms range. HRV feature extraction followed ultra-short-term analysis standards (Shaffer and Ginsberg, 2017) and included three domains: (1) time-domain metrics, (2) frequency-domain metrics, and (3) nonlinear metrics (Table 2). Frequency-domain analyses were conducted on overlapping temporal windows, and 120-s segments were identified as providing the best balance between signal stability and temporal resolution. For HRV parameters of SDNN, RMSSD, pNN50, LF and HF power, four summary statistics (mean, standard deviation, minimum, maximum) were computed across the task period. Additionally, to prevent the model from learning spurious correlations based on absolute magnitude (e.g., “larger values indicate higher score of emotion”), we prioritized bias-resistant features: (1) individual-normalized features that standardize each participant’s data to their own baseline, (2) detrended features that remove temporal trends, (3) ratio and coefficient of variation features that capture relative patterns rather than absolute scales, and (4) variability measures (e.g., standard deviation of HRV) that reflect physiological dynamics. These statistical measures served as scalar features for modeling.

**TABLE 2:** Summary of HRV parameters.

Type	Parameter	Description
Time domain measures	SDNN	Standard deviation of NN intervals (time interval between successive heartbeats)
	RMSSD	Root mean square of successive RR differences
	pNN50	Percentage of successive RR intervals (interbeat intervals between all successive heartbeats) that differ by more than 50 ms
Frequency domain measures	LF power	Absolute power of low-frequency band (0.04–0.15 Hz)
	HF power	Absolute power of high-frequency band (0.15–0.4 Hz)
	LF/HF	Ratio of LF-to-HF power
Non-linear measures	SD1	Poincaré plot standard deviation perpendicular the line of identity
	SD2	Poincaré plot standard deviation along the line of identity
	SD1/SD2	Ratio of SD1-to-SD2

For each participant, baseline signals were subtracted from the task data (baseline correction), and the result was then min–max scaled. This two-step normalization procedure enhanced cross-participant comparability and proved particularly important for maintaining HRV signal stability. Finally, we optimized data padding and interpolation strategies by replacing simple forward-fill interpolation with stretch-based linear interpolation (Supplementary Appendix D). This approach yielded more continuous physiological time series and improved classification accuracy across all physiological modalities, including EDA, HRV, and their fusion models.

#### 2.6 LSTM-based deep learning for physiological signal modeling

Two complementary streams of inputs were produced from the preprocessing steps of EDA and HRV signals: (1) multichannel, temporally standardized EDA/HRV sequences and (2) scalar feature vectors comprising EDA- and HRV-derived summary statistics. We implemented both input streams (i.e., the time-series, sequential stream, and the engineered scalar stream) in our model to capture fine-grained temporal dynamics and global physiological trends. Long Short-Term Memory (LSTM) neural networks were employed to model time series as they are well suited for capturing long-range temporal dependencies that characterize biological signals (Pham, 2021). The time-series stream processes the interpolated EDA/HRV channels standardized to 300 timesteps under three configurations: (1) EDA-only, (2) HRV-only, or (3) multimodal fusion of EDA and HRV (see details in Supplementary Appendix E). These inputs span specific temporal windows: either the task period plus 2-min recovery (for EDA and fusion models) or from 5 min pre-task through 2 min post-task (for the HRV model, where individual normalization requires a baseline reference). This temporal refinement was designed to account for the lagged dynamics of autonomic activity relative to emotional stimuli, improving the temporal alignment between physiological measures and affective events. This stream utilizes two stacked LSTM layers followed by a dense layer with 12 ReLU units. To examine the trade-off between model complexity and performance, we explored two configurations for the LSTM layers: a wide structure (with 64 and 32 units, respectively) and a narrow structure (48 and 24 units).

**FIGURE 2:**
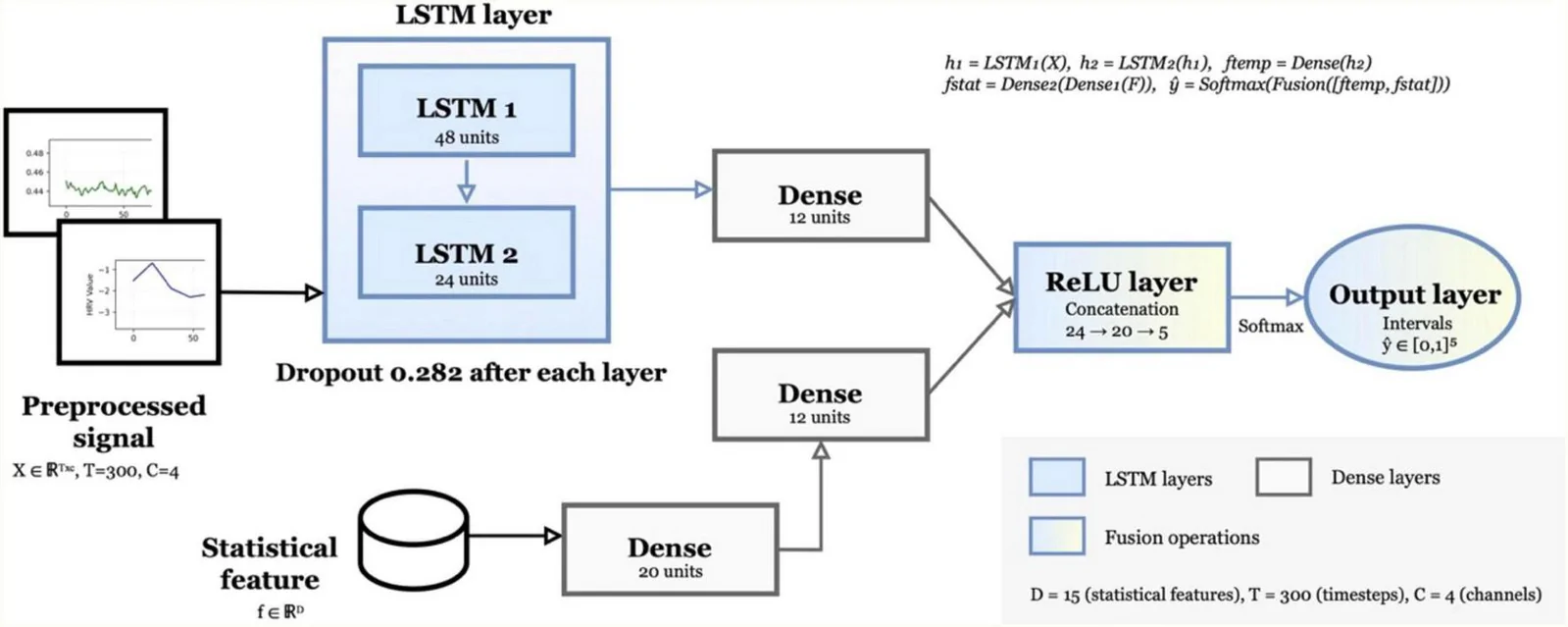
Best-performing LSTM architecture integrating EDA and HRV time-series with engineered statistical features.

The scalar stream receives all the engineered summary features derived from EDA and HRV signals (descriptive statistics and bias-resistant features) and complements the sequence modeling with global information. This stream has two dense layers with 20 and 12 ReLU units. The outputs from both streams are then concatenated and passed to a final fusion head, which consists of a 20-unit ReLU layer, a dropout layer, and a Softmax layer for five-class classification. Figure 2 illustrates this two-stream fusion architecture.

Models were trained for up to 300 epochs with a batch size of 16 using the Adam optimizer and sparse categorical cross-entropy loss. Key hyperparameters were tuned to optimize performance; the learning rate was tested in the range of 0.0005–0.01, and dropout rates were manually adjusted between 0.15 and 0.4 to balance model fit and regularization against persistent overfitting. We implemented several callbacks to manage the training process: early stopping (patience = 25), learning rate reduction on plateau (factor = 0.5, patience = 8), and model checkpointing to save the best-performing model. To ensure robust and reproducible results, all experiments were conducted with a fixed random seed (42), a consistent preprocessing and evaluation pipeline. The models were implemented with TensorFlow 2.16.0-rc0 (Keras 3.9.2) in Python 3.9.6. To provide a more conservative estimate of the models’ performance, we evaluated them using participant-level (rather than clip-level) 5-fold cross-validation for two reasons. First, the underlying goal of this study is to test whether individuals’ physiological signals predict their own emotion ratings, rather than to predict ratings for unseen video clips; participant-level grouping therefore matches this goal, as it evaluates generalization across observers rather than across stimuli. Second, because the current 20-clip corpus is relatively small, clip-level grouping would yield unstable estimates. Accordingly, in each fold, all observations from the same participant were assigned to the same split, ensuring no participant overlap between training and testing data. After physiological signal quality control, 1,747 of the 1,760 raw participant-clip observations were retained for LSTM modeling; observations with incomplete or invalid EDA/HRV recordings were excluded.

## 3 Results

### 3.1 Human ratings

Eighty-eight bilingual raters evaluated the 20 courtroom clips (10 Chinese, 10 English) on a 21-point valence scale (0 = strongly negative, 10 = neutral, 20 = strongly positive). Clip means ranged from 1.26 to 10.08, indicating a predominantly negative-to-neutral stimulus set (Table 3). Across the 20 clips, 1 clip fell in the [10–20] range (positive), 9 clips (5 Chinese, 4 English) in the [8–10) range (neutral-leaning), 3 clips (1 Chinese, 2 English) in the [6–8) range (slightly negative), 2 clips (2 Chinese) in the [4–6) range (moderately negative), 3 clips (1 Chinese, 2 English) in the [2–4) range (very negative), and 2 clips (2 English) in the [0–2) range (strongly negative).

**TABLE 3:** Summary of human valence ratings for courtroom video clips.

Case ID	Language	Rating (mean)	SD	Coefficient of variation	ICC
Case 1a	Chinese	8.864	2.117	0.240	0.874
Case 1b	Chinese	8.227	3.136	0.383	0.890
Case 2a	Chinese	8.727	2.614	0.301	0.877
Case 2b	Chinese	4.045	3.802	0.945	0.838
Case 3a	Chinese	9.784	1.133	0.116	0.936
Case 3b	Chinese	5.250	4.071	0.780	0.738
Case 4a	Chinese	9.580	1.776	0.186	0.911
Case 4b	Chinese	2.875	2.628	0.919	0.769
Case 5a	Chinese	10.080	2.175	0.217	0.941
Case 5b	Chinese	7.602	3.722	0.492	0.845
Case 6a	English	8.625	2.647	0.309	0.913
Case 6b	English	2.875	4.405	1.541	0.804
Case 7a	English	9.375	2.846	0.305	0.918
Case 7b	English	7.045	3.649	0.521	0.851
Case 8a	English	9.068	5.584	0.619	0.685
Case 8b	English	1.409	2.219	1.584	0.862
Case 9a	English	9.614	2.940	0.308	0.913
Case 9b	English	2.841	2.872	1.017	0.666
Case 10a	English	7.398	3.153	0.429	0.889
Case 10b	English	1.261	2.014	1.606	0.864

SD, standard deviation; ICC, Intraclass correlation coefficient.

Variability across clips was moderate, with standard deviations ranging from 1.13 to 5.58 and coefficients of variation from 0.12 to 1.61. Some clips, particularly strongly negative English stimuli, exhibited high relative variability (coefficients of variation > 1), indicating greater divergence in rater perceptions. Inter-rater reliability (ICC, two-way random, absolute agreement) ranged from 0.666 to 0.941, indicating overall good to excellent agreement. Chinese clips generally showed higher ICCs (0.74–0.94), whereas English clips exhibited more variability across raters (0.67–0.92). These rating distributions and reliability estimates constitute the human reference against which computational, psychometric, and physiological models are evaluated.

### 3.2 Sentiment analysis

Two complementary evaluation metrics were employed to assess tool performance: categorical alignment accuracy, measuring the proportion of exact band matches between machine-predicted sentiment categories and human ratings, and Spearman’s rank correlation coefficients, capturing the extent to which machine outputs preserved the relative ordering of human sentiment ratings (see Supplementary Appendix F for converted outputs). Shapiro-Wilk tests confirmed that the human ratings, averaged across participants for each of the 20 clips, deviated significantly from normality (*W* = 0.853, *p* = 0.006), warranting the use of non-parametric Spearman’s rank correlations, given the bounded rating scale and the focus on rank-order correspondence.

The combination of categorical and correlational analyses revealed a marked dissociation: overall categorical accuracy remained low, whereas several tools achieved strong rank correlations with human ratings (Table 4). Across tools, performance was consistently stronger for Chinese courtroom materials than for English materials, though this advantage manifested differently across metrics. Chinese texts achieved a mean categorical accuracy of 0.49, compared to 0.21 for English texts, with an overall average of 0.35. Of the seven tools that processed both languages, five showed higher categorical accuracy for Chinese, one showed equal accuracy across languages, and one favored English. The mean paired advantage for Chinese over English was 0.24, 95% CI [−0.01, 0.50], Cohen’s dz = 0.88 [paired *t*(6) = 2.33, *p* = 0.06]. The cross-linguistic gap is thus a large but with considerable uncertainty. At the item level, predictions for Chinese clips were more tightly clustered and consistent across analytical frameworks, whereas English clip predictions were more dispersed, reflecting broader variability both within and between tools. Spearman correlation analyses mirrored this pattern: tools generally aligned more closely with human ratings for Chinese texts than for English (Table 4).

**TABLE 4:** Alignment between machine sentiment analysis tools and human ratings.

Tool	Accuracy	No. aligned (ZH) (/10)	ρ (ZH) (95% CI)	p (raw) (ZH)	p (BH) (ZH)	No. aligned (EN) (/10)	ρ (EN) (95% CI)	p (raw) (EN)	p (BH) (EN)
GPT-4o	0.20	1	0.8842 (0.57, 0.97)	0.0007	0.0029	3	0.9598 (0.83, 0.99)	< <0.0001	0.0002
DeepSeek-R1	0.20	2	0.8602 (0.50, 0.97)	0.0014	0.0042	2	0.8789 (0.56, 0.97)	0.0008	0.0029
BERT-GO-Emotions	0.45	7	0.8020 (0.35, 0.95)	0.0053	0.0105	2	0.0935 (–0.57, 0.68)	0.7973	0.8441
BERT-Multilingual	0.00	–	–	–	–	0	0.1508 (–0.53, 0.71)	0.6775	0.7622
VADER	0.30	–	–	–	–	3	0.1758 [–0.51, 0.73]	0.6272	0.7525
TextBlob	0.20	–	–	–	–	2	0.5212 (–0.16, 0.87)	0.1224	0.2002
HanLP	0.30	3	–0.2705 (–0.77, 0.43)	0.4497	0.5782	–	–	–	–
Google Cloud NL API	0.55	6	0.8980 (0.62, 0.98)	0.0004	0.0025	5	0.8362 (0.44, 0.96)	0.0026	0.0058
Amazon Comprehend	0.40	6	0.9211 (0.69, 0.98)	0.0002	0.0014	2	0.4660 (–0.23, 0.85)	0.1747	0.2418
Amazon Rekognition	0.35	6	0.8431 (0.46, 0.96)	0.0022	0.0056	1	0.0000 (–0.63, 0.63)	1.0000	1.0000
Microsoft Azure Video Indexer	0.40	6	0.7143 (0.15, 0.93)	0.0203	0.0365	2	0.5060 (–0.18, 0.86)	0.1356	0.2035

ZH, Chinese; EN, English. “No. aligned” indicates the number of clips for which the machine-predicted emotion band exactly matched the human-consensus emotion band, out of 10 clips in each language. Dashes indicate that the relevant tool did not support or was not applied to that language. The 95% confidence intervals are unadjusted and describe the uncertainty surrounding Spearman’s ρρ. p (raw) = unadjusted two-sided *p*-value; p (BH) = Benjamini–Hochberg-adjusted *p*-value.

Individual tool performance exhibited substantial variability. LLMs demonstrated strong correlations with human mean ratings (ρ = 0.86–0.96) (Table 4), though their categorical accuracy remained relatively low, suggesting high sensitivity to sentiment intensity and directionality but lower precision in discrete categorization. Google Cloud Natural Language API (cloud-based NLP service) achieved the highest categorical accuracy (0.55) alongside strong correlations, reflecting more balanced performance. By contrast, language-specific models underperformed relative to expectations: HanLP exhibited a negative correlation with human ratings (ρ = −0.27) for Chinese texts; several lexicon- and classifier-based tools for English had CIs spanning zero (VADER; BERT-Multilingual; BERT-GO-Emotions; Amazon Rekognition), indicating unreliable rank alignment. Non-textual tools demonstrated intermediate performance: Amazon Rekognition and Microsoft Azure Video Indexer achieved moderate categorical accuracies (0.35 and 0.40, respectively) and generally weaker correlations for English clips.

Importantly, agreement among sentiment analysis tools was consistently low. Fleiss’ kappa analyses (see Supplementary Appendix G) yielded low or negative values (κ = −0.20 to −0.11) across sentiment analysis tools for 19 out of the 20 video clips. As a robustness check for class imbalance, Gwet’s AC1 was also computed; agreement remained low for both textual tools, AC1 = 0.18, and the full tool set, AC1 = 0.19, supporting the conclusion of limited cross-tool convergence. These results indicate limited convergence among tools, particularly under the skewed distribution of emotion categories in the present dataset. The sole exception was one Chinese case (3a), which showed perfect agreement (κ = 1.00), but this was isolated and atypical.

### 3.3 The prediction performance of the psychometric measurement

**FIGURE 3:**
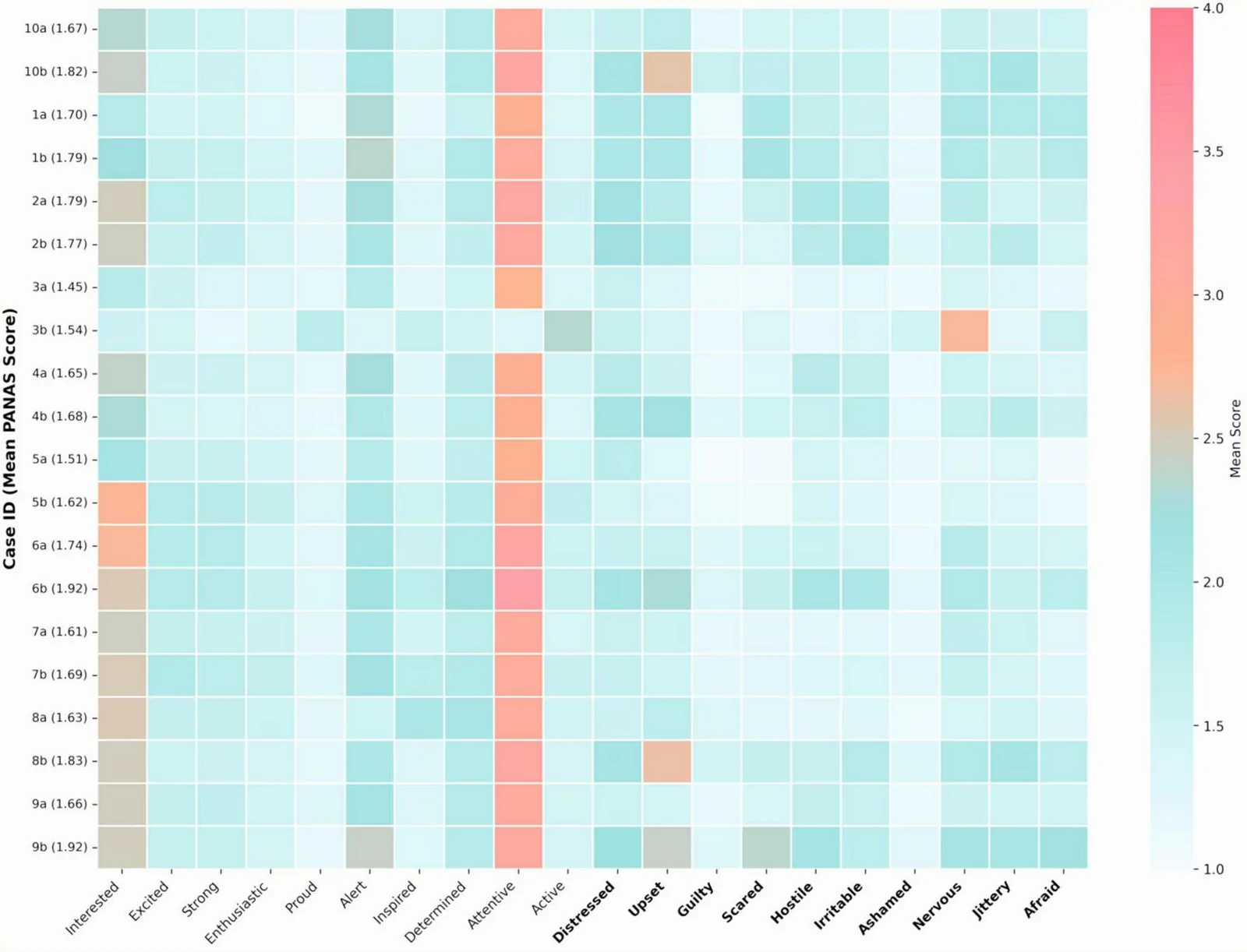
Heatmap of mean PANAS scores by task (Negative emotions in bold).

Figure 3 visualizes mean PANAS scores across tasks. The heatmap reveals a pronounced engagement profile: Attentive is consistently the highest-intensity state across nearly all tasks, with Interested also elevated. Among negative effects, Upset and Nervous show the clearest peaks and variability, followed by more modest elevations in Distressed, whereas other emotions remain comparatively muted. Overall, the map exhibits a narrow valence spread—few extremes beyond Attentive—suggesting constrained dispersion in self-reports. This pattern implies that the stimuli chiefly elicit cognitive engagement with selective increases in Upset and Nervous rather than broad activation of fear, guilt, or shame.

**FIGURE 4:**
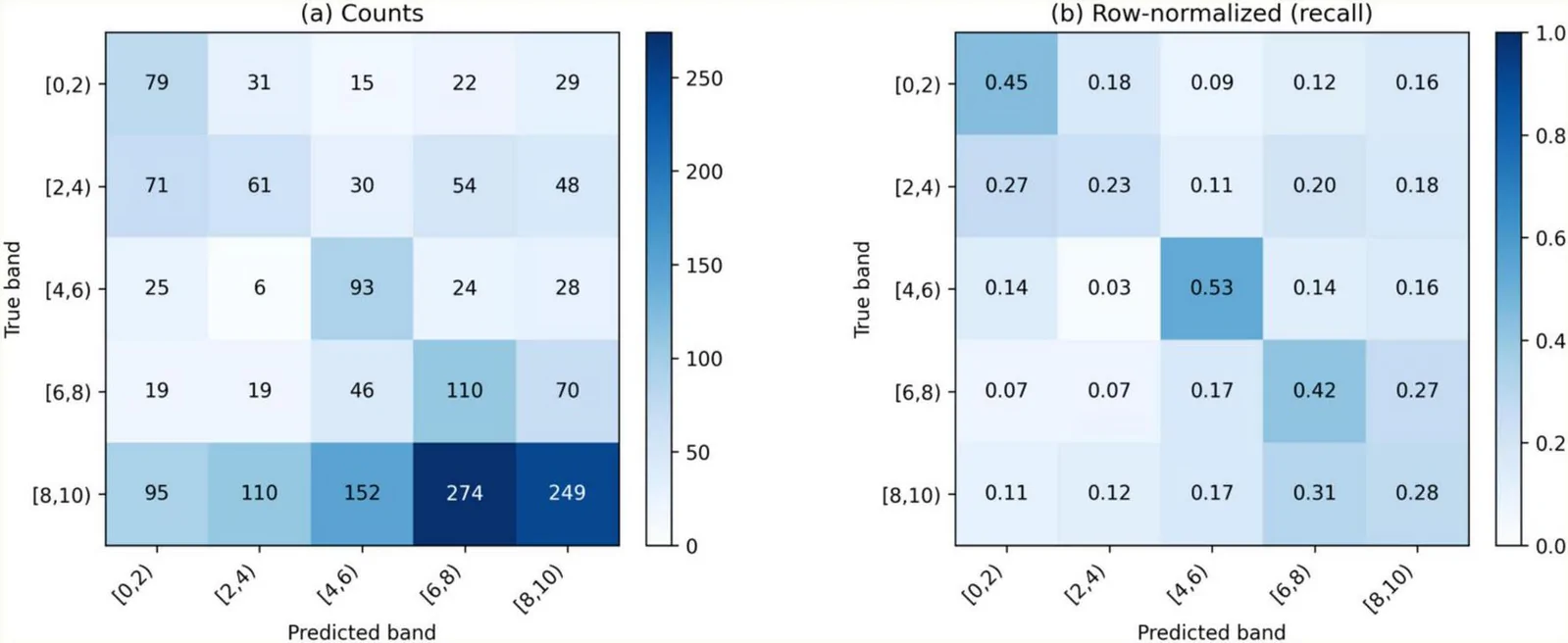
Confusion matrix of PANAS logistic regression (20 individual items) under participant-level 5-fold cross-validation.

Table 5 reports logistic regression performance on PANAS scores under participant-level 5-fold cross-validation. The best-performing configuration, using the 20 individual items, still reached only 0.336 accuracy (balanced accuracy = 0.382, macro-F1 = 0.326). The confusion matrix (Figure 4) shows misclassifications distributed across nearly all class pairs rather than concentrated along the diagonal. Class-specific recall was similarly uneven, ranging from 0.231 for [2–4) to 0.528 for [4–6), with [0–2), [6–8), and [8–10) falling in between at 0.449, 0.417, and 0.283, respectively. Together, these results indicate that PANAS self-reports captured little of the variance in observers’ perceived-emotion labels.

**TABLE 5:** Logistic regression performance using PANAS feature sets.

No.	Feature set	Features (k)	Accuracy	Balanced accuracy	Macro-F1
1	Positive and negative total scores	2	0.223	0.287	0.217
2	Individual items	**20**	**0.336**	**0.382**	**0.326**
3	Positive emotions	10	0.205	0.276	0.199
4	Negative emotions	10	0.268	0.353	0.276

Bold values indicate the best-performing configuration in the table.

### 3.4 Prediction performance of physiological measures

#### 3.4.1 Overall results

Most LSTM-based models using physiological data achieved moderate performance. Accuracy ranged from 0.668 to 0.773 across models, with several configurations exceeding 0.70 (Table 6). The EDA and HRV all-feature fusion model reached the highest numerical accuracy [0.773 ± 0.085, 95 % CI (0.698, 0.847)], but its confidence interval overlapped with several HRV-based models, which precludes a definitive claim of statistical superiority. Among single-modality models, HRV models generally performed better than the EDA model. HRV time-domain plus non-linear features and HRV all features performed comparably well, while fusing EDA with HRV produced only limited improvement. Even the weakest configurations, i.e., EDA alone and HRV frequency-domain plus non-linear features, still exceeded the majority-class baseline, indicating that both signals carry some usable affective information.

**TABLE 6:** LSTM performance by modality and feature set (mean ± SD across runs).

LSTM model	Accuracy (95% CI)	Balanced accuracy (95% CI)	Macro-F1 (95% CI)	Architecture	Hyperparameter
EDA	0.668 ± 0.014 (0.656, 0.680)	0.513 ± 0.019 (0.496, 0.530)	0.493 ± 0.031 (0.466, 0.520)	64→32 (LSTM layers) → 16 (dense layer)	Dropout: 0.3, learning rate 0.001
HRV (All-feature)	0.750 ± 0.072 (0.688, 0.813)	0.640 ± 0.119 (0.535, 0.745)	0.649 ± 0.134 (0.532, 0.766)	48→24 (LSTM layers) →12 (dense layer)	Dropout: 0.282, learning rate 0.01
HRV (Time domain + non-linear measures)	0.756 ± 0.069 (0.695, 0.817)	0.662 ± 0.107 (0.568, 0.756)	0.677 ± 0.131 (0.562, 0.792)	64→32 (LSTM layers) →16 (dense layer)	Dropout: 0.2, learning rate 0.001
HRV (Frequency domain + non-linear measures)	0.677 ± 0.029 (0.652, 0.703)	0.523 ± 0.032 (0.495, 0.551)	0.504 ± 0.045 (0.464, 0.543)	64→32 (LSTM layers) →16 (dense layer)	Dropout: 0.2, learning rate 0.01
EDA + HRV (All-feature)	**0.773 ± 0.085 (0.698, 0.847)**	**0.680 ± 0.123 (0.572, 0.788)**	**0.686 ± 0.141 (0.563, 0.810)**	48→24 (LSTM layers) →12 (dense layer)	Dropout: 0.282, learning rate 0.01
EDA + HRV (Time domain + non-linear measures)	0.681 ± 0.053 (0.634, 0.728)	0.540 ± 0.093 (0.458, 0.622)	0.531 ± 0.122 (0.425, 0.638)	64→32 (LSTM layers) →16 (dense layer)	Dropout: 0.25, learning rate 0.00075
EDA + HRV (Frequency domain + non-linear measures)	0.709 ± 0.067 (0.651, 0.768)	0.609 ± 0.119 (0.505, 0.713)	0.604 ± 0.138 (0.484, 0.725)	64→32 (LSTM layers) →16 (dense layer)	Dropout: 0.3, learning rate 0.001

Bold values indicate the best-performing configuration in the table.

#### 3.4.2 Detailed performance analysis of the final physiological model

**FIGURE 5:**
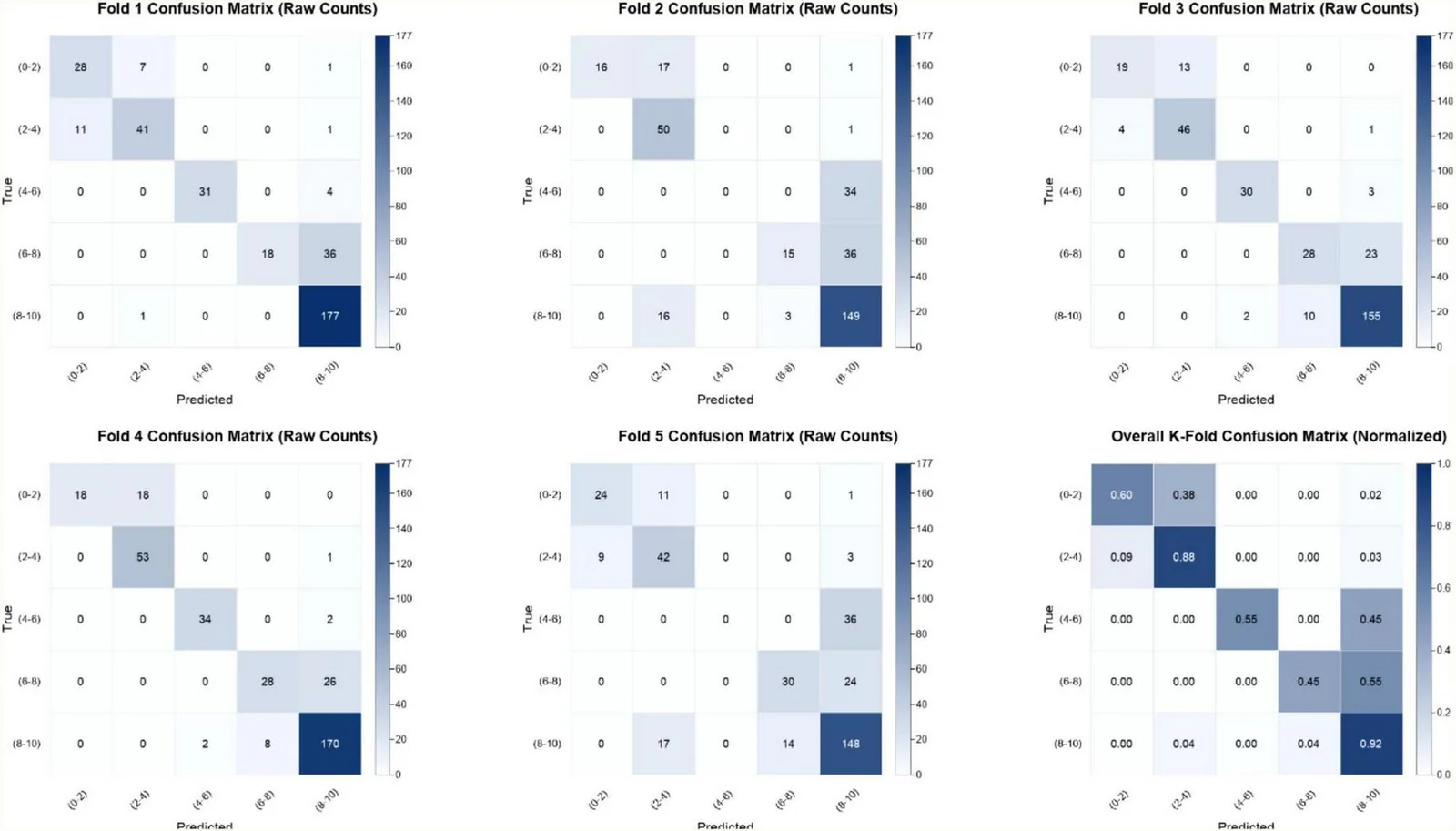
Confusion matrix for the final model (EDA + HRV all data) by raw counts of samples across participant-level 5-fold validation and overall normalized.

Based on the physiological model comparisons, the multimodal LSTM model using combined EDA and all HRV features was retained as the final physiological model under participant-level five-fold cross validation (see Table 6). Examination of the confusion matrices for this model (see Figure 5) reveals distinct patterns in classification across emotion rating intervals. The consistency of these patterns across 5-fold cross-validation suggests that they reflect stable, underlying properties of the physiology-emotion mapping rather than random variability.

The model excelled at identifying states with distinct physiological signatures. The very negative [2–4) and neutral [8–10) categories were classified with pooled recalls at 0.88 and 0.92, respectively, indicating clear and consistent physiological markers for these states. Performance was moderate for [0–2) and [4–6), with recalls of 0.60 and 0.55, and weakest for [6–8), with recall of 0.45. Most errors for the intermediate [4–6) and [6–8) categories were shifted toward the [8–10) category, suggesting that mildly negative or moderately negative responses were more difficult to separate from near-neutral responses. This likely reflects the unbalanced dataset, in which the neutral [8–10) category made up around half of the data, giving the model ample data to learn the features for the neutral state, but comparatively few examples of the more negative states. Even so, the negative categories were far from unlearnable: the model still achieved 0.630 ± 0.120 accuracy when distinguishing between the four negative categories alone. This suggests that these classes carry learnable physiological signal despite their limited representation.

## 4 Discussion

This study offers a multimodal evaluation of observer-perceived emotion recognition in cross-linguistic legal contexts, comparing computational sentiment analysis, psychometric self-reports, and physiological measures across Chinese and English courtroom materials. The findings highlight different characteristics of the tested modalities: sentiment analysis tools provide scalable but variable estimates, PANAS captures broad observer engagement but shows weak predictability for observers’ perceived-emotion labels, and physiological indicators, particularly the EDA and HRV fusion LSTM model, showed relatively commendable predictive performance under participant-level cross validation, although their performance remains limited by class imbalance and corpus size. These findings highlight both the potential of physiological measures to enhance emotion recognition in high-stakes legal settings and the persistent challenges of achieving cross-linguistic comparability, extending beyond the well-known limitations of traditional sentiment analysis. The multimodal framework developed here underscores the complementary strengths and weaknesses of different approaches and provides a methodological foundation for advancing emotion research in professional and culturally diverse contexts.

### 4.1 Physiological signals capture the emotional substrate of courtroom interactions

The participant-level physiological analysis suggests that observer autonomic responses contain information relevant to perceived emotional expression in formal legal settings. Courtroom interactions are characterized by strategic emotional displays, suppression, and professional composure (Maroney, 2011; Terry, 2008), which can make surface cues difficult to interpret. By capturing observers’ autonomic nervous system activity, physiological methods may offer an additional layer of insight into affective resonance with courtroom content. This result is consistent with the emotional contagion/congruence framework (Hatfield et al., 1993; Barsade, 2002), suggesting that observers’ physiological states may shift in alignment with the affective tone of courtroom content they witness.

The methodological backbone of this approach lies in the use of LSTM networks, which are well suited to modeling the temporal dependencies inherent in physiological time series (Pham, 2021). Unlike traditional classifiers that process independent segments, LSTMs preserve information over extended sequences, allowing emotional patterns that unfold gradually to be captured more effectively. To capture complementary autonomic dynamics, we fused EDA—a marker of sympathetic arousal (Boucsein, 2012; Critchley, 2002)—with HRV features, which reflect parasympathetic regulation (Thayer et al., 2012; Shaffer and Ginsberg, 2017). The HRV feature set encompassed time-domain, frequency-domain, and non-linear measures, providing a rich quantitative profile of autonomic activity (Olivieri et al., 2024). This complementary combination allows simultaneous tracking of stress-induced arousal and regulatory control, two key physiological systems engaged during high-stakes courtroom interactions.

Under participant-level cross validation, the fusion of EDA and HRV model achieved accuracy of 0.773, balanced accuracy of 0.680, and macro-F1 of 0.686. These results indicate a promising signal in the physiological stream, and align with recent work showing that integrating multiple physiological channels enhances emotion recognition performance (Boffet et al., 2025; Nandini et al., 2025), though the present corpus size and observer-based proxy design warrant conservative claims about generalizability. However, improvement over the strongest HRV-based models was modest, and their confidence intervals overlapped. In the present architecture, representations learned from the physiological sequence stream and the engineered-feature stream were combined through fixed concatenation before classification, which preserves both temporally evolving and global physiological information; but it does not adaptively change the relative contribution of the input streams. Fixed concatenation captured complementary information but did not demonstrate clear superiority over HRV-based modeling. In broader multimodal recognition research, gated fusion has been adopted to learn input-dependent weights for different modalities, particularly when the signals differ in informativeness or temporal alignment (Ahmad et al., 2025). Future comparisons with gated fusion could determine whether adaptive weighting improves EDA–HRV integration.

Looking beyond model comparisons, however, these findings suggest that physiological measures offer an informative window into observers’ affective responses to courtroom contents, capturing autonomic activity that may be less dependent on explicit self-report or textual expression. In legal contexts, where emotional expression is often restrained or strategically managed, autonomic measures (EDA and HRV, whether combined or used separately) provide a useful complementary indicator of observer-perceived affective dynamics.

### 4.2 The paradox of sentiment analysis in legal emotion recognition

The performance of computational sentiment analysis tools revealed a striking paradox: while individual tools showed strong alignment with human judgments (i.e., high rank correlations), they exhibited systematically poor agreement with one another. LLMs demonstrated exceptionally high correlations with human ratings, consistent with recent findings that LLMs can capture linguistic nuance and contextual emotional meaning with remarkable sensitivity (Guo et al., 2024). These high correlations suggest that models trained on massive, diverse corpora can recognize emotional expression in legal discourse with considerable sophistication, leveraging their exposure to varied communicative contexts and emotional registers.

Yet, this individual strength contrasts sharply with the lack of consensus between tools. Across the set of sentiment analysis systems, Fleiss’ kappa values ranged from –0.20 to –0.11, indicating limited convergence among tools. These divergences are not random; they arise from systematic differences in how models represent and detect affect. Architectural choices influence which linguistic and contextual cues are prioritized; training data shape the emotional norms and label distributions to which models are calibrated; and each system’s implicit operationalization of affect—often encoded in its annotation schema and optimization objective—helps define the boundary between “emotionally salient” and “neutral” input (Chang et al., 2024; Song et al., 2024; Poria et al., 2020). Consequently, rather than uniformly failing to detect emotion, these systems may be capturing different but equally valid emotional cues, depending on their analytical frameworks.

Legal discourse, characterized by low-arousal affect, irony, indirectness, and layered pragmatic meaning, further amplifies these divergences by pushing different analytical frameworks to prioritize distinct textual signals. Such properties of legal language have long been recognized in legal linguistics (Danet, 1980; Cotterill, 2003) and pose significant challenges for domain adaptation in NLP (Ashley, 2017). This jagged technological frontier reflects an uneven landscape in which tools excel in different niches but fail to converge on a shared interpretive baseline.

The divergence between analytical frameworks becomes particularly apparent when comparing broad, general-purpose models with more narrowly focused tools. Cloud-based enterprise NLP services, particularly Google Cloud Natural Language API, achieved the highest categorical accuracy (0.55), likely benefiting from extensive, systematically curated training data and iterative refinement (Goyal et al., 2024). By contrast, the performance of specialized sentiment analysis tools was unexpectedly poor. Language-optimized systems such as HanLP (ρ = –0.2705) and VADER (ρ = 0.1758) underperformed relative to general-purpose transformer-based and cloud models, suggesting that narrow task-specific tuning may not generalize well to the specialized register of legal discourse. Non-textual visual tools, such as Amazon Rekognition and Microsoft Azure Video Indexer, showed intermediate performance, capturing broad affective tendencies with moderate categorical accuracy but aligning less consistently with human valence judgments in complex legal settings. These results indicate that breadth of linguistic competence and domain coverage, rather than narrow optimization for a particular language or sentiment lexicon, may offer a more robust foundation for capturing the emotional complexity and nuance of legal discourse (Zhang et al., 2024).

Overall, models trained on diverse, large-scale datasets (e.g., LLMs and general-purpose APIs) outperformed lexicon-based or language-specific alternatives in the current study. However, agreement between tools was low, revealing that while individual state-of-the-art models can approximate human judgments, they achieved it via divergent and often incompatible ways. Thus, methodological convergence across systems has yet to be achieved in the field. This fragmentation raises critical questions about standardization, validity, and interpretability in computational emotion recognition for legal applications, and highlights the need for integrative frameworks capable of reconciling these analytical differences.

### 4.3 Self-reported affect captures broad engagement but lacks discriminative power

The participant-level PANAS analysis highlights both the utility and limitations of self-report measures for emotion recognition in legal contexts. PANAS logistic regression reached only weak imbalance-sensitive performance (best balanced accuracy = 0.382; macro-F1 = 0.326). As a broad valence–arousal instrument (Watson et al., 1988), PANAS is well suited to capturing general affective engagement, but it is not optimized for fine-grained emotion bands discrimination. Observers tended to assign extreme ratings to a subset of emotionally salient clips while offering less differentiated responses to subtler displays, thereby reducing alignment with the observer-perceived emotional-expression labels. This suggests that PANAS may be sensitive to high-arousal events but insufficient for detecting how observers perceive the more nuanced affective cues that characterize courtroom interactions.

Additionally, because the instrument was administered post hoc, it likely captured integrated affective impressions rather than moment-by-moment tracking. This aligns with evidence that observer self-reports index their own emotional experience, which can diverge substantially from the emotional content of the stimulus (Scherer, 2005; Barrett et al., 2019). In legal settings, where emotional displays are often restrained or strategically modulated, observers may experience tension, moral unease, or empathy that does not map cleanly onto the actors’ expressed emotions.

Another contributing factor is the individual variability in emotional conceptualization and labeling. Research shows that people differ in the granularity and stability of their emotion representations, leading to systematic differences in how they interpret identical emotional displays (Barrett, 2004; Keating and Cook, 2023). This heterogeneity likely contributes to the observed class imbalance and moderate accuracy, as some observers provide consistently coarse or extreme ratings, while others discriminate more finely. Such variability underscores the introspective and interpretive nature of PANAS reporting.

Overall, these findings indicate that while self-report measures like PANAS can provide valuable insights into observers’ global affective engagement, they offer limited discriminative power for recognizing discrete emotions in complex, strategically managed legal contexts. Their strength may lie in indexing broad affective states, not in delivering precise emotion labels. As such, PANAS self-reports should be viewed as complementary to physiological and computational modalities rather than as a stand-alone benchmark for emotion recognition.

### 4.4 Cross-linguistic variation reveals asymmetries in emotional processing and modeling

A striking pattern across both human ratings and sentiment analysis tools was the stronger performance or agreement on Chinese courtroom materials than on English materials. This asymmetry likely reflects a combination of factors, including observers’ native-language background, differences between the Chinese and English legal systems and communicative norms, and variation in recording platforms and corpus sources. Part of the gap may also be a measurement artifact rather than a purely linguistic one: Chinese clips showed higher inter-rater reliability than English clips (Table 3), and since tool accuracy is scored against these ratings as ground truth, a noisier reference standard for English could suppress apparent agreement regardless of tool quality. Language, corpus source, and rating reliability co-vary across our 20 clips and cannot be fully disentangled here. Even so, these convergent human–machine patterns across two independent measures suggests that at least part of the asymmetry reflects genuine difficulty in cross-linguistic emotion recognition, where subtle affective cues are shaped by language, legal culture, and communicative context.

On the human side, raters exhibited higher inter-rater reliability when evaluating Chinese materials, and tended to judge them as more emotionally neutral overall. In contrast, English materials—particularly those containing negative emotional content—elicited greater variability and lower agreement, even though all raters possessed advanced English proficiency and the materials were subtitled. This pattern aligns with research on bilingual emotional processing, which shows that emotional interpretation in L2 is often less automatic, less intense, and more variable than in the native language (L1), even at high proficiency levels (Caldwell-Harris, 2014; Pavlenko, 2012). For the present participants, Chinese courtroom discourse may therefore provide more familiar linguistic and cultural cues. Emotional nuance, cultural connotations, and subtle affective cues are often more robustly represented and processed in the L1 (Chinese), where they are embedded in early-learned, embodied emotional networks, whereas L2 (English) processing tends to be more cognitively mediated and less emotionally resonant, leading to greater variability in perceived affect.

Computational analyses revealed parallel asymmetries. Sentiment analysis tools performed markedly better on Chinese legal texts (49% categorical accuracy) than on English materials (21%). This difference does not necessarily indicate superior NLP performance for Chinese per se; rather, it likely reflects a relative alignment between the stylistic features of legal Chinese and the training distributions of the tools, compared to the more complex and pragmatically nuanced register of legal English. Legal discourse in both languages is characterized by formality, ritualized phrasing, and an institutional presumption of neutrality (Danet, 1980). However, legal English often employs indirect or layered pragmatic strategies (e.g., sarcasm, hedging, implicit threats), which can confuse models trained primarily on informal or explicit emotional expressions from social media and product reviews. By contrast, legal Chinese involve more lexically and syntactically regularized formulations (cf. Han et al., 2018; Zhang, 2021), which may effectively minimize the domain mismatch between legal texts and standard training data, allowing generic models to process the more predictable Chinese legal register with greater accuracy than its English counterpart.

These convergent cross-linguistic patterns underscore that both human raters and computational models exhibit language-specific processing biases that influence emotion recognition. For human raters, these biases arise from deeply rooted bilingual emotional asymmetries; for sentiment analysis models, from training corpus mismatches and linguistic register differences. In legal settings, such asymmetries are far from trivial: they can affect the reliability of emotional interpretation, potentially introducing systematic biases across languages in contexts where emotional nuance—such as sarcasm, remorse, or defiance—can influence judgments and outcomes. Addressing these challenges requires not only careful consideration of raters’ linguistic and cultural backgrounds, but also domain-adaptive training of NLP models to handle the distinctive affective registers of legal discourse in multiple languages.

### 4.5 Implications for legal practice and methodology

Beyond their contribution to affective computing and psycholinguistics, these findings carry concrete implications for legal practice and forensic methodology. Emotion is not incidental to legal proceedings: it shapes witness credibility assessments, influences juror decision-making, and affects perceptions of procedural fairness, yet legal systems currently lack validated, objective tools for capturing affective dynamics in courtroom interactions. The present study suggests that observer-based physiological paradigms may serve as a methodologically tractable complement to text-based and self-report approaches, especially where direct monitoring of courtroom actor is ethically and logistically impractical. For legal practitioners and policymakers, the findings urge caution about uncritical deployment of off-the-shelf computational sentiment tools in legal contexts—whether for analyzing testimony, assessing defendant affect, or evaluating judicial communication—given the systematic domain mismatch demonstrated here. The cross-linguistic asymmetries further warn that tools validated in one legal language or jurisdiction may perform poorly in another, a consideration of particular importance in multilingual legal systems and international tribunals. Taken together, these results call for the development of domain-adapted, culturally sensitive emotion-recognition frameworks specifically validated on legal discourse, and for methodological standards in forensic affective research that require reporting inter-tool agreement alongside individual model performance.

### 4.6 Limitations and future directions

Several limitations should be acknowledged. First, observer ratings capture the affect that courtroom actors express and that observers perceive, not the actors’ subjective internal states. Although this design is grounded in emotional-contagion theory and supported by good-to-excellent inter-rater reliability, the resulting labels reflect observer consensus about displayed affect and may diverge from what courtroom actors actually felt; they should therefore be read as an observer-perceived emotional-expression benchmark rather than as direct evidence of actors’ emotional experience. Second, although the PANAS and physiological models were evaluated at the participant-clip observation level, the corpus still contains only 20 unique courtroom clips; generalization across legal genres, languages, and jurisdictions therefore remains limited. This small stimulus set also means that, although the cross-validation design ensures subject-independent robustness (no participant appears in both training and test folds, per section 2.6), it does not ensure stimulus-independent robustness, since the same 20 clips necessarily recur across folds, rated by different participants. Model performance should therefore be interpreted as generalizing across observers of a fixed stimulus set rather than to unseen courtroom footage. Third, class imbalance was substantial, with the [8–10) band accounting for approximately half of the physiological observations, making balanced accuracy, F1-score, and class-wise recall essential for interpretation. Fourth, because sentiment analysis, PANAS, and physiological measures draw on fundamentally different sources of information (text, subjective self-report, and autonomic signal, respectively) and were evaluated under different units of analysis, observed differences in performance across modalities should not be read as evidence of one approach’s intrinsic methodological superiority over another. Finally, the Chinese and English clips were drawn from different platforms and legal systems (China Court Trial Online vs. YouTube channels), so the cross-linguistic contrasts may be confounded with corpus source, recording conditions, and jurisdiction, and should be read as exploratory rather than as evidence of inherent language differences. Larger, more balanced, multi-site corpora with matched recording conditions and fully nested validation are needed before these findings can support applied forensic decision-making.

## 5 Conclusion

This exploratory study demonstrates that computational sentiment analysis tools, PANAS self-reports, and physiological measures each capture a partially distinct, non-redundant slice of observer-perceived affect in legal discourse. Physiological models showed the most consistent signal: the EDA and HRV fusion LSTM reached the highest numerical accuracy (0.773) in predicting observer-perceived emotion labels, indicating that the autonomic signals carry promising information for modeling observer-perceived affect in legal settings. While large language models and cloud-based NLP tools showed strong correlation with observer ratings, their reliability was undermined by low inter-tool agreement and cross-linguistic performance variability, reflecting divergent analytical frameworks and domain mismatches. Similarly, self-reported affect provided insights into broad observer emotional engagement but lacked the discriminative precision needed to capture fine-grained, subtle, strategically managed emotions.

These preliminary results position multimodal, culturally sensitive, and validation-transparent approaches as a promising foundation for future research on emotion recognition in legal contexts. Beyond their methodological contribution, these findings open new avenues for studying emotional contagion, observer resonance, and affective dynamics in high-stakes decision-making settings, while also underscoring the need for larger matched datasets before emotion-recognition tools are deployed in applied judicial contexts.

## Data Availability

The datasets and supporting materials presented in this study are available in Zenodo at: https://zenodo.org/records/21494375.

## Footnotes

1
https://www.court.gov.cn/tingshen.html

2
http://www.ledalab.de

## References

[B1] Ahmad B. Khan M. Sajjad M. (2025). Gated fusion networks for multi-modal violence detection. *AI* 6:259. 10.3390/ai6100259

[B2] Althubaiti A. (2016). Information bias in health research: Definition, pitfalls, and adjustment methods. *J. Multidiscip. Healthc.* 9 211–217. 10.2147/JMDH.S104807 27217764 PMC4862344

[B3] Ash E. Chen D. L. Galletta S. (2022). Measuring judicial sentiment: Methods and application to US circuit courts. *Economica* 89 362–376. 10.1111/ecca.12397

[B4] Ashley K. D. (2017). *Artificial Intelligence and Legal Analytics: New Tools for Law Practice in the Digital Age.* Cambridge: Cambridge University Press. 10.1017/9781316761380

[B5] Bandes S. A. Blumenthal J. A. (2012). Emotion and the law. *Annu. Rev. Law Soc. Sci.* 8 161–181. 10.1146/annurev-lawsocsci-102811-173825

[B6] Barrett L. F. (2004). Feelings or words? Understanding the content in self-report ratings of experienced emotion. *J. Pers. Soc. Psychol.* 87 266–281. 10.1037/0022-3514.87.2.266 15301632 PMC1351136

[B7] Barrett L. F. Adolphs R. Marsella S. Martinez A. M. Pollak S. D. (2019). Emotional expressions reconsidered: Challenges to inferring emotion from human facial movements. *Psychol. Sci. Public Interest* 20 1–68. 10.1177/1529100619832930 31313636 PMC6640856

[B8] Barsade S. G. (2002). The ripple effect: Emotional contagion and its influence on group behavior. *Adm. Sci. Q.* 47 644–675. 10.2307/3094912

[B9] Benedek M. Kaernbach C. (2010). A continuous measure of phasic electrodermal activity. *J. Neurosci. Methods* 190 80–91. 10.1016/j.jneumeth.2010.04.028 20451556 PMC2892750

[B10] Boffet A. Arsac L. M. Ibanez V. Sauvet F. Deschodt-Arsac V. (2025). Detection of cognitive load modulation by EDA and HRV. *Sensors* 25:2343. 10.3390/s25082343 40285033 PMC12030397

[B11] Boucsein W. (2012). *Electrodermal Activity.* Berlin: Springer Science & Business Media.

[B12] Caldwell-Harris C. L. (2014). Emotionality differences between a native and foreign language: Theoretical implications. *Front. Psychol.* 5:1055. 10.3389/fpsyg.2014.01055 25295019 PMC4172089

[B13] Chang Y. Wang X. Wang J. Wu Y. Yang L. Zhu K. et al. (2024). A survey on evaluation of large language models. *ACM Trans. Intell. Syst. Technol.* 15 1–45. 10.1145/3641289

[B14] Cotterill J. (2003). *Language and Power in Court: A Linguistic Analysis of the OJ Simpson Trial.* Berlin: Springer.

[B15] Critchley H. D. (2002). Electrodermal responses: What happens in the brain. *Neuroscientist* 8 132–142. 10.1177/107385840200800209 11954558

[B16] Danet B. (1980). Language in the legal process. *Law Soc. Rev.* 14 445–564. 10.2307/3053192

[B17] Ekman P. (1992). An argument for basic emotions. *Cogn. Emot.* 6 169–200. 10.1080/02699939208411068

[B18] Farias D. H. Rosso P. (2017). “Irony, sarcasm, and sentiment analysis,” in *Sentiment Analysis in Social Networks*, eds Pozzi F. A. Fersini E. Messina E. Liu B. (Boston, MA: Morgan Kaufmann), 113–128. 10.1016/B978-0-12-804412-4.00007-3

[B19] Gashi S. Di Lascio E. Santini S. (2019). Using unobtrusive wearable sensors to measure the physiological synchrony between presenters and audience members. *Proc. ACM Interact. Mob. Wearable Ubiquitous Technol.* 3 1–19. 10.1145/3314400

[B20] Gerboni G. Comunale G. Chen W. Lever Taylor J. Migliorini M. Picard R. et al. (2023). Prospective clinical validation of the Empatica EmbracePlus wristband as a reflective pulse oximeter. *Front. Digit. Health* 5:1258915. 10.3389/fdgth.2023.1258915 38111608 PMC10726006

[B21] Gong L. Chen W. Li M. Zhang T. (2024). Emotion recognition from multiple physiological signals using intra-and inter-modality attention fusion network. *Digit. Signal Proc.* 144:104278. 10.1016/j.dsp.2023.104278

[B22] Goyal S. Hussein F. Thukral I. Trimble T. (2024). “Empowering enterprise architecture: Leveraging NLP for time efficiency and strategic alignment,” in *Proceedings of the 2024 Systems and Information Engineering Design Symposium (SIEDS)*, (Charlottesville, VA: IEEE), 431–436. 10.1109/SIEDS61124.2024.10534749

[B23] Greenwald A. G. Banaji M. R. (1995). Implicit social cognition: Attitudes, self-esteem, and stereotypes. *Psychol. Rev.* 102 4–27. 10.1037/0033-295x.102.1.4 7878162

[B24] Guo R. Guo H. Wang L. Chen M. Yang D. Li B. (2024). Development and application of emotion recognition technology—a systematic literature review. *BMC Psychol.* 12:95. 10.1186/s40359-024-01581-4 38402398 PMC10894494

[B25] Han B. Yau C. Lei S. Gratch J. (2024). “Knowledge-based emotion recognition using large language models,” in *Proceedings of the 2024 12th International Conference on Affective Computing and Intelligent Interaction (ACII)*, (Glasgow: IEEE), 1–9. 10.1109/ACII63134.2024.00005

[B26] Han Z. Bhatia V. K. Ge Y. (2018). The structural format and rhetorical variation of writing Chinese judicial opinions: A genre analytical approach. *Pragmatics* 28 463–487. 10.1075/prag.17013.ge

[B27] Hatfield E. Cacioppo J. T. Rapson R. L. (1993). Emotional contagion. *Curr. Dir. Psychol. Sci.* 2 96–100. 10.1111/1467-8721.ep10770953

[B28] Hess U. Fischer A. (2013). Emotional mimicry as social regulation. *Pers. Soc. Psychol. Rev.* 17 142–157. 10.1177/1088868312472607 23348982

[B29] Huang L. Yang T. Li Z. (2003). Applicability of the positive and negative affect scale in Chinese. *Chin. Ment. Health J.* 17 54–56.

[B30] Jiang Y. Li W. Hossain M. S. Chen M. Alelaiwi A. Al-Hammadi M. (2020). A snapshot research and implementation of multimodal information fusion for data-driven emotion recognition. *Inf. Fusion* 53 209–221. 10.1016/j.inffus.2019.06.019

[B31] Kamble K. Sengupta J. (2023). A comprehensive survey on emotion recognition based on electroencephalograph (EEG) signals. *Multimed. Tools Appl.* 82 27269–27304. 10.1007/s11042-023-14489-9

[B32] Keating C. T. Cook J. L. (2023). The inside out model of emotion recognition: How the shape of one’s internal emotional landscape influences the recognition of others’ emotions. *Sci. Rep.* 13:21490. 10.1038/s41598-023-48469-8 38057460 PMC10700588

[B33] Khare S. K. Blanes-Vidal V. Nadimi E. S. Acharya U. R. (2024). Emotion recognition and artificial intelligence: A systematic review (2014–2023) and research recommendations. *Inf. Fusion* 102:102019. 10.1016/j.inffus.2023.102019

[B34] Kojima T. Gu S. S. Reid M. Matsuo Y. Iwasawa Y. (2022). Large language models are zero-shot reasoners. *Adv. Neural Inf. Process. Syst.* 35 22199–22213. 10.52202/068431-1613

[B35] Konvalinka I. Roepstorff A. (2012). The two-brain approach: How can mutually interacting brains teach us something about social interaction? *Front. Hum. Neurosci.* 6:215. 10.3389/fnhum.2012.00215 22837744 PMC3402900

[B36] Levenson R. W. (2011). Basic emotion questions. *Emot. Rev.* 3 379–386. 10.1177/1754073911410743

[B37] Liu P. Yuan W. Fu J. Jiang Z. Hayashi H. Neubig G. (2023). Pre-train, prompt, and predict: A systematic survey of prompting methods in natural language processing. *ACM Comput. Surv.* 55 1–35. 10.1145/3560815

[B38] Maroney T. A. (2006). Law and emotion: A proposed taxonomy of an emerging field. *Law Hum. Behav.* 30 119–142. 10.1007/s10979-006-9029-9 16786403

[B39] Maroney T. A. (2011). Emotional regulation and judicial behavior. *Calif. Law Rev.* 99 1485–1555. 10.15779/Z38XQ3J

[B40] Mehrabian A. (1980). *Basic Dimensions for a General Psychological Theory: Implications for Personality, Social, Environmental, and Developmental Studies.* Cambridge, MA: Oelgeschlager, Gunn & Hain.

[B41] Michalec B. Forbes C. E. Pardon K. Ayala B. Beltran D. G. Douille C. et al. (2025). A scoping review of emotional contagion research with human subjects: Identifying common trends of previous research and potential areas for future research. *Front. Psychol.* 16:1573375. 10.3389/fpsyg.2025.1573375 40519824 PMC12164912

[B42] Mohammad S. Turney P. (2010). “Emotions evoked by common words and phrases: Using mechanical turk to create an emotion lexicon,” in *Proceedings of the NAACL HLT 2010 Workshop on Computational Approaches to Analysis and Generation of Emotion in Text*, Los Angeles, CA, 26–34.

[B43] Munezero M. Montero C. S. Sutinen E. Pajunen J. (2014). Are they different? Affect, feeling, emotion, sentiment, and opinion detection in text. *IEEE Trans. Affect. Comput.* 5 101–111. 10.1109/TAFFC.2014.2317187

[B44] Nandini D. Yadav J. Singh V. Mohan V. Agarwal S. (2025). An ensemble deep learning framework for emotion recognition through wearable devices multi-modal physiological signals. *Sci. Rep.* 15:17263. 10.1038/s41598-025-99858-0 40383809 PMC12086232

[B45] Olivieri F. Biscetti L. Pimpini L. Pelliccioni G. Sabbatinelli J. Giunta S. (2024). Heart rate variability and autonomic nervous system imbalance: Potential biomarkers and detectable hallmarks of aging and inflammaging. *Ageing Res. Rev.* 101:102521. 10.1016/j.arr.2024.102521 39341508

[B46] Ouyang L. Wu J. Jiang X. Almeida D. Wainwright C. Mishkin P. et al. (2022). Training language models to follow instructions with human feedback. *Adv. Neural Inf. Process. Syst.* 35 27730–27744.

[B47] Palumbo R. V. Marraccini M. E. Weyandt L. L. Wilder-Smith O. McGee H. A. Liu S. et al. (2017). Interpersonal autonomic physiology: A systematic review of the literature. *Pers. Soc. Psychol. Rev.* 21 99–141. 10.1177/1088868316628405 26921410

[B48] Pang B. Lee L. (2008). Opinion mining and sentiment analysis. *Found. Trends Inf. Retr.* 2 1–135. 10.1561/1500000011

[B49] Pavlenko A. (2012). Affective processing in bilingual speakers: Disembodied cognition? *Int. J. Psychol.* 47 405–428. 10.1080/00207594.2012.743665 23163422

[B50] Pham T. D. (2021). Time-frequency time-space LSTM for robust classification of physiological signals. *Sci. Rep.* 11:6936. 10.1038/s41598-021-86432-7 33767352 PMC7994826

[B51] Picard R. W. (2000). *Affective Computing.* Cambridge, MA: MIT Press. 10.7551/mitpress/1140.001.0001

[B52] Plutchik R. (1980). “A general psychoevolutionary theory of emotion,” in *Theories of Emotion*, eds Plutchik R. Kellerman H. (New York, NY: Academic Press), 3–33. 10.1016/B978-0-12-558701-3.50007-7

[B53] Poria S. Cambria E. Bajpai R. Hussain A. (2017). A review of affective computing: From unimodal analysis to multimodal fusion. *Inf. Fusion* 37 98–125. 10.1016/j.inffus.2017.02.003

[B54] Poria S. Hazarika D. Majumder N. Mihalcea R. (2020). Beneath the tip of the iceberg: Current challenges and new directions in sentiment analysis research. *IEEE Trans. Affect. Comput.* 14 108–132. 10.1109/TAFFC.2020.3038167

[B55] Prochazkova E. Kret M. E. (2017). Connecting minds and sharing emotions through mimicry: A neurocognitive model of emotional contagion. *Neurosci. Biobehav. Rev.* 80 99–114. 10.1016/j.neubiorev.2017.05.013 28506927

[B56] Rokach L. (2010). Ensemble-based classifiers. *Artif. Intell. Rev.* 33 1–39. 10.1007/s10462-009-9124-7

[B57] Russell J. A. (1980). A circumplex model of affect. *J. Pers. Soc. Psychol.* 39 1161–1178. 10.1037/h0077714

[B58] Salerno J. M. Peter-Hagene L. C. (2015). One angry woman: Anger expression increases influence for men, but decreases influence for women, during group deliberation. *Law Hum. Behav.* 39 581–592. 10.1037/lhb0000147 26322952

[B59] Šandor D. Babac M. B. (2023). Sarcasm detection in online comments using machine learning. *Inf. Discov. Deliv.* 52 213–226. 10.1108/IDD-01-2023-0002

[B60] Scherer K. R. (2005). What are emotions? And how can they be measured? *Soc. Sci. Inf.* 44 695–729. 10.1177/0539018405058216

[B61] Shaffer F. Ginsberg J. P. (2017). An overview of heart rate variability metrics and norms. *Front. Public Health* 5:258. 10.3389/fpubh.2017.00258 29034226 PMC5624990

[B62] Song S. He C. Li S. Zhao S. Wang C. Yan T. et al. (2024). MOSABench: Multi-object sentiment analysis benchmark for evaluating multimodal large language models understanding of complex image. *ArXiv [Preprint]* 10.48550/arXiv.2412.00060

[B63] Sorin V. Brin D. Barash Y. Konen E. Charney A. Nadkarni G. et al. (2024). Large language models and empathy: Systematic review. *J. Med. Internet Res.* 26:e52597. 10.2196/52597 39661968 PMC11669866

[B64] Sunshine J. Tyler T. R. (2003). The role of procedural justice and legitimacy in shaping public support for policing. *Law Soc. Rev.* 37 513–547. 10.1111/1540-5893.3703002

[B65] Szoszkiewicz Ł. Yuste R. (2025). Mental privacy: Navigating risks, rights and regulation: Advances in neuroscience challenge contemporary legal frameworks to protect mental privacy. *EMBO Rep.* 26 3469–3473. 10.1038/s44319-025-00505-6 40562792 PMC12287510

[B66] Tao J. Tan T. Picard R. W. (2007). “Affective computing and intelligent interaction,” in *Proceedings of the 2nd International Conference, ACII*, Lisbon.

[B67] Terry L. S. (2008). The future regulation of the legal profession: The impact of treating the legal profession as service providers. *J. Prof. Lawyer* 2008 189–212.

[B68] Thayer J. F. Åhs F. Fredrikson M. Sollers J. J. III Wager T. D. (2012). A meta-analysis of heart rate variability and neuroimaging studies: Implications for heart rate variability as a marker of stress and health. *Neurosci. Biobehav. Rev.* 36 747–756. 10.1016/j.neubiorev.2011.11.009 22178086

[B69] Tyler T. R. (2006). Psychological perspectives on legitimacy and legitimation. *Annu. Rev. Psychol.* 57 375–400. 10.1146/annurev.psych.57.102904.190038 16318600

[B70] Warriner A. B. Kuperman V. Brysbaert M. (2013). Norms of valence, arousal, and dominance for 13,915 English lemmas. *Behav. Res. Methods* 45 1191–1207. 10.3758/s13428-012-0314-x 23404613

[B71] Watson D. Clark L. A. Tellegen A. (1988). Development and validation of brief measures of positive and negative affect: The PANAS scales. *J. Pers. Soc. Psychol.* 54 1063–1070. 10.1037//0022-3514.54.6.1063 3397865

[B72] Zhang F. (2021). *A Comparative Study of Chinese and Western Legal Language and Culture.* Singapore: Springer. 10.1007/978-981-15-9347-5

[B73] Zhang W. Deng Y. Liu B. Pan S. Bing L. (2024). “Sentiment analysis in the era of large language models: A reality check,” in *Findings of the Association for Computational Linguistics: NAACL 2024*, eds Duh K. Gomez H. Bethard S. (Mexico: Association for Computational Linguistics), 3881–3906. 10.18653/v1/2024.findings-naacl.246

[B74] Zhu L. Mou W. Hong C. Yang T. Lai Y. Qi C. et al. (2024). The evaluation of generative AI should include repetition to assess stability. *JMIR MHealth UHealth* 12:e57978. 10.2196/57978 38688841 PMC11106698

