# Supplementary material for: Emotion recognition in cross-linguistic legal context: a comparison between human-based and computational approaches
Source: Front Psychol. 2026 Sep 3;17:1859697. doi: 10.3389/fpsyg.2026.1859697 (PMC13581832; doi:10.3389/fpsyg.2026.1859697)
Supplement: Supplementary file 1 [file Table_1.docx]

**Appendices**

**Appendix A: Details of material difficulty control**

**Table 7** Textual difficulty metrics of all materials

| **Language** | **Case no.** | **Word**  **/character count** | **Word**  **/character TTR** | **Content word density** | **Propositional density** | **Flesch reading ease score** | **Deliver speed (words/characters per minute)** |
| --- | --- | --- | --- | --- | --- | --- | --- |
| Chinese | 1a | 154 | 0.57 | 0.59 | 0.447 | n/a | 181.176 |
|  | 1b | 164 | 0.62 | 0.63 | 0.666 | n/a | 164.000 |
|  | 2a | 142 | 0.61 | 0.56 | 0.429 | n/a | 181.277 |
|  | 2b | 153 | 0.55 | 0.38 | 0.375 | n/a | 103.146 |
|  | 3a | 140 | 0.59 | 0.73 | 0.358 | n/a | 161.538 |
|  | 3b | 147 | 0.51 | 0.37 | 0.462 | n/a | 154.737 |
|  | 4a | 152 | 0.59 | 0.35 | 0.428 | n/a | 165.818 |
|  | 4b | 156 | 0.59 | 0.23 | 0.690 | n/a | 156.000 |
|  | 5a | 155 | 0.65 | 0.30 | 0.459 | n/a | 178.846 |
|  | 5b | 149 | 0.61 | 0.35 | 0.512 | n/a | 109.024 |
| English | 6a | 159 | 0.55 | 0.40 | 0.509 | 80.4 | 180.000 |
|  | 6b | 155 | 0.54 | 0.43 | 0.497 | 76.6 | 120.779 |
|  | 7a | 149 | 0.66 | 0.44 | 0.544 | 57.7 | 159.643 |
|  | 7b | 136 | 0.62 | 0.37 | 0.529 | 83.0 | 125.538 |
|  | 8a | 164 | 0.52 | 0.32 | 0.537 | 78.0 | 144.706 |
|  | 8b | 157 | 0.58 | 0.40 | 0.497 | 82.3 | 122.338 |
|  | 9a | 164 | 0.60 | 0.41 | 0.549 | 61.3 | 189.231 |
|  | 9b | 156 | 0.65 | 0.54 | 0.538 | 72.0 | 146.250 |
|  | 10a | 149 | 0.65 | 0.43 | 0.544 | 65.1 | 175.294 |
|  | 10b | 153 | 0.67 | 0.44 | 0.510 | 79.1 | 150.492 |

Remarks for Table 7:

For English materials, word count and propositional density were calculated using CPIDR 5 (Covington, 2009). Word TTR was calculated using the Wordlist module in AntConc software. Materials were first tagged by the English USAS (UCREL Semantic Analysis System) semantic tagger (Rayson et al., 2004) using the CLAWS7 tag set (<https://ucrel.lancs.ac.uk/claws7tags.html>), and content words were counted using the Concordance module in AntConc according to the CLAWS7 tag set. Flesch reading ease score (Kincaid et al., 1975) was also used to assess difficulty, though it is only available for English materials.

In terms of Chinese materials, character count, character TTR, word count, and content word density were calculated using a Chinese text grading system developed by Peking University (see <http://www.chinese-pku.com>). Propositions were analyzed using HanLP (He & Choi, 2021) with the Chinese propositional bank 3.0 (Xue et al., 2013), and the number of propositions was calculated manually. Since propositions were analyzed at the word level (which may contain multiple characters) according to the propositional bank, propositional density was proportionally scaled based on character count to ensure comparability with English.

**Table 8** Descriptive statistics of Chinese vs. English materials’ textual difficulty

| **Materials** | **Metrics** | **Word**  **/character count** | **Word**  **/character TTR** | **Content word density** | **Propositional density** | **Flesch reading ease score** | **Deliver speed (words/characters per minute)** |
| --- | --- | --- | --- | --- | --- | --- | --- |
| Chinese | Mean | 152.89 | 0.5894 | 0.4333 | 0.5014 | n/a | 152.45 |
|  | SD | 8.01 | 0.0436 | 0.1279 | 0.0853 | n/a | 26.54 |
| English | Mean | 151.00 | 0.6600 | 0.4350 | 0.5270 | 73.60 | 162.89 |
|  | SD | 2.83 | 0.0141 | 0.0071 | 0.0240 | 9.12 | 17.54 |

**References**

Covington, M. A. (2009). Idea density—A potentially informative characteristic of retrieved documents. In *IEEE Southeastcon 2009* (pp. 201-203). IEEE.

He, H., & Choi, J. D. (2021). The stem cell hypothesis: Dilemma behind multi-task learning with transformer encoders. In *Proceedings of the 2021 Conference on Empirical Methods in Natural Language Processing* (pp. 5555-5577).

Kincaid, J. P., Fishburne Jr, R. P., Rogers, R. L., & Chissom, B. S. (1975). Derivation of new readability formulas (automated readability index, fog count and flesch reading ease formula) for navy enlisted personnel.

Rayson, P., Archer, D. E., Piao, S. L., & McEnery, T. (2004). The UCREL semantic analysis system. In *Proceedings of the workshop on beyond named entity recognition semantic labelling for nlp tasks, in association with lrec-04* (pp. 7-12). European Language Resources Association.

Xue, N., Bai, X., Jill, L., Zhang, J., Palmer, M., Chang, M., & Zhong, H. (2013). *Chinese proposition bank 3.0 LDC2013T13*. Linguistic Data Consortium. https://doi.org/10.35111/828e-w727

**Appendix B: Prompting procedures, preprocessing steps, and parameter settings regarding GPT-4o and DeepSeek-R1**

**Prompting procedures**

For the large-language-model sentiment analysis, 20 selected courtroom transcript excerpts (10 English and 10 Chinese) were analyzed using GPT-4o and DeepSeek-R1. Only the transcript text was submitted to the models; no audio or video information was included.

Each excerpt was analyzed separately in three independent runs for each model. Every run began in a new conversation and used the same two-step prompting sequence. The first prompt requested an overall emotional-valence classification and confidence estimate:

“*Please help me analyze the sentiment/emotion of the following texts. You should give me the emotional valence of the text, Negative-Neutral-Positive, and your estimation confidence score. Because the texts are extracted from authentic courts, you just need to evaluate the emotions of the speaker according to the text. You do not need to judge whether the behavior is positive or negative in the context, such as criminal acts, illegal behaviors, evidence lists, or case outcomes. Text: [transcript excerpt].*”

A follow-up prompt was then used to obtain a more fine-grained estimate of emotional valence. This prompt asked the model to report the relative degree of positive, negative, and neutral emotion and to provide an overall valence bias:

“*Can you detail the degree of the emotional valence? For example, how much is positive, how much is negative, how much is neutral. What is its overall bias? Base your estimate strictly on the speaker's expressed or implied emotional tone, not on the legal severity, culpability, or outcome of the case.*”

This procedure yielded 120 independent model conversations: 20 excerpts × 2 models × 3 runs. Because each conversation contained two prompt submissions, the complete procedure comprised 240 individual prompt submissions.

**Model-interface settings**

GPT-4o was accessed through the ChatGPT public interface using the default settings available at the time of analysis. DeepSeek-R1 was accessed through its public interface using the default reasoning mode, with web search disabled. No model-specific sampling parameters were manually adjusted. Neither model was queried through an API or automated script.

**Appendix C: Nested cross-validation and dimensionality-reduction robustness check (PANAS)**

To verify that the participant-clip results were not driven by the fixed regularization used in Table 5, we repeated the analysis on the same 1,760 participant-clip observations (88 observers × 20 clips) with fully nested cross-validation. The outer loop was a subject-independent 5-fold GroupKFold (grouped by observer); within each outer training partition, an inner 4-fold subject-grouped loop selected the regularization strength (C ∈ {0.01, 0.1, 1, 10}), the penalty (L1/L2), and an optional dimensionality-reduction step (none, PCA, or univariate SelectKBest) by maximizing balanced accuracy, so that no test fold influenced model selection.

**Table 9** Logistic regression performance using nested cross-validation and dimensionality-reduction

| **Feature set** | **k** | **Accuracy** | **Balanced accuracy** | **Macro-F1** | **Clip probability accuracy** | **Selected configuration (inner loop)** |
| --- | --- | --- | --- | --- | --- | --- |
| Individual items | 20 | 0.338 | **0.380** | 0.325 | 0.500 | L2; C = 0.1 (4/5 folds); none or full-rank PCA/SKB(18) |
| Positive + Negative totals | 2 | 0.222 | 0.286 | 0.217 | 0.450 | L2; C = 0.01–10; no reduction (4/5 folds) |
| Positive items | 10 | 0.200 | 0.276 | 0.197 | 0.350 | L2; C = 0.1–10; no reduction (all folds) |
| Negative items | 10 | 0.254 | 0.343 | 0.264 | 0.350 | L2; C = 0.01–10; PCA/SKB(5–8) |

*Notes:* **a)** Clip probability accuracy: observation-level out-of-fold class probabilities are averaged across the ~88 observers of each clip (soft voting) and the clip is labelled by the highest averaged probability; the value is the proportion of the 20 clips matching their observer-consensus band. This column is clip-level (N = 20) for comparability with the clip-based tools; all other columns are observation-level (N = 1,760). **b)** Selected configuration summarizes the penalty, regularization strength (C), and dimensionality-reduction step chosen by the inner loop across the five outer folds (per-fold selections in the replication package). All models used class-balanced logistic regression.

Nested tuning did not materially change performance (see Fig 6). The best-performing feature set—the 20 individual PANAS items—reached balanced accuracy = 0.380 (accuracy = 0.338, macro-F1 = 0.325), essentially identical to the fixed-hyperparameter model reported in Table 5 (accuracy = 0.336, balanced accuracy = 0.382, macro-F1 = 0.326). Subscale-only sets remained weaker (negative items = 0.343; positive/negative totals = 0.286; positive items = 0.276). Across outer folds the inner loop most often retained L2 regularization with little or no dimensionality reduction, indicating that neither PCA nor univariate selection improved fine-grained discrimination. These results confirm that the limited five-class discriminability of PANAS reflects the signal in the self-reports rather than hyperparameter overfitting.

**Fig. 6** Confusion matrix of the best nested-CV configuration (Individual items model) with subject-independent 5-fold cross-validation

**Appendix D: Effects of interpolation padding**

**Fig. 7** Interpolated data samples

**Appendix E: Details of model channels/features**

**Table 10** Model streams of different configurations

| **Configuration** | **Stream** | **Primitive channel(s)/Features** | **Data input** | **Bias resistant processing** |
| --- | --- | --- | --- | --- |
| EDA only | Sequence stream | 1 channel | EDA raw data time series | Interpolation padding |
|  | Scalar stream | 10 features | EDA raw data: Mean/Std/Max/Min  SCR amplitude: Count/Mean/Std/Max/Min  Task duration (to tag data according to stimuli presentation) | Baseline normalization  Detrended processing  Coefficient of variation and relative range calculation |
| HRV only | Sequence stream | 3 channels | HRV time series: Mean/Std/Max/Min of mean HR, RMSSD, LF/HF ratio | Interpolation padding |
|  | Scalar stream | 41 features | Mean/Std/Max/Min of Mean HR, SDNN, RMSSD, pNN50, SD1, SD2, SD ratio, LF power, HF power, LF/HF ratio  Task duration (to tag data according to stimuli presentation) | Baseline normalization  Detrended processing  Mean HR/SDNN ratio calculation |
| Fusion of EDA and HRV | Sequence stream | 4 channels | The combination of EDA only and HRV only | Interpolation padding |
|  | Scalar stream | 51 features | The combination of EDA only and HRV only | The combination of EDA only and HRV only |

| **Task** | **Legal context** | **GPT-4o** | **Deepseek-R1** | **Google Cloud NL API** | **Amazon Comprehend** | **BERT-GO-Emotions** | **BERT-Multilingual** | **VADER** | **TextBlob** | **HanLP** | **Microsoft Azure** | **Amazon Rekognition** |
| --- | --- | --- | --- | --- | --- | --- | --- | --- | --- | --- | --- | --- |
| Case 1a | Chinese | [4-6) | [4-6) | [8-10) | [8-10) | [8-10) | N/A | N/A | N/A | [10+) | [8-10) | (8-10) |
| Case 1b | Chinese | [0-2) | [0-2) | [6-8) | [8-10) | [8-10) | N/A | N/A | N/A | [10+) | [0-2) | (8-10) |
| Case 2a | Chinese | [6-8) | [6-8) | [8-10) | [8-10) | [8-10) | N/A | N/A | N/A | [8-10) | [8-10) | (8-10) |
| Case 2b | Chinese | [0-2) | [0-2) | [4-6) | [2-4) | [2-4) | N/A | N/A | N/A | [10+) | [0-2) | (8-10) |
| Case 3a | Chinese | [8-10) | [8-10) | [8-10) | [8-10) | [8-10) | N/A | N/A | N/A | [8-10) | [8-10) | (8-10) |
| Case 3b | Chinese | [2-4) | [2-4) | [6-8) | [2-4) | [2-4) | N/A | N/A | N/A | [10+) | [0-2) | (8-10) |
| Case 4a | Chinese | [6-8) | [6-8) | [8-10) | [8-10) | [8-10) | N/A | N/A | N/A | [8-10) | [8-10) | (8-10) |
| Case 4b | Chinese | [0-2) | [0-2) | [4-6) | [6-8) | [4-6) | N/A | N/A | N/A | [10+) | [2-4) | (8-10) |
| Case 5a | Chinese | [6-8) | [8-10) | [8-10) | [8-10) | [8-10) | N/A | N/A | N/A | [10+) | [8-10) | (8-10) |
| Case 5b | Chinese | [2-4) | [2-4) | [6-8) | [4-6) | [6-8) | N/A | N/A | N/A | [6-8) | [8-10) | (8-10) |
| Case 6a | English | [6-8) | [0-2) | [6-8) | [0-2) | [8-10) | [6-8) | [2-4) | [10+) | N/A | [0-2) | (10+) |
| Case 6b | English | [2-4) | [0-2) | [6-8) | [6-8) | [10+) | [10+) | [2-4) | [8-10) | N/A | [0-2) | (0-2) |
| Case 7a | English | [6-8) | [6-8) | [8-10) | [4-6) | [10+) | [10+) | [10+) | [8-10) | N/A | [6-8) | (10+) |
| Case 7b | English | [4-6) | [0-2) | [6-8) | [10+) | [4-6) | [4-6) | [6-8) | [8-10) | N/A | [8-10) | (8-10) |
| Case 8a | English | [6-8) | [4-6) | [4-6) | [2-4) | [10+) | [4-6) | [10+) | [8-10) | N/A | [2-4) | (8-10) |
| Case 8b | English | [0-2) | [0-2) | [0-2) | [0-2) | [10+) | [6-8) | [0-2) | [10+) | N/A | [0-2) | (10+) |
| Case 9a | English | [6-8) | [6-8) | [10+) | [10+) | [10+) | [10+) | [10+) | [10+) | N/A | [10+) | (8-10) |
| Case 9b | English | [0-2) | [0-2) | [2-4) | [0-2) | [2-4) | [0-2) | [0-2) | [6-8) | N/A | [0-2) | (10+) |
| Case 10a | English | [6-8) | [2-4) | [8-10) | [6-8) | [8-10) | [10+) | [2-4) | [8-10) | N/A | [0-2) | (8-10) |
| Case 10b | English | [2-4) | (0-2) | [8-10) | [10+) | [10+) | [10+) | [10+) | [10+) | N/A | [2-4) | (8-10) |

**Appendix F: Converted outputs from computational tools**

**Table 11** Converted outputs from computational tools

**Appendix G: Cross-tool Fleiss’ kappa for each case and robustness check**

Fleiss’ kappa and Gwet’s AC1 were computed using NumPy (2.1.3) and Pandas (2.2.3) in Python 3.10.15, following their standard formulas.

**Table 12** Fleiss’ kappa results

| **Case ID** | **Language** | **Textual Fleiss κ** | **Overall Fleiss κ** |
| --- | --- | --- | --- |
| Case 1a | Chinese | -0.20 | -0.14 |
| Case 1b | Chinese | -0.20 | -0.14 |
| Case 2a | Chinese | -0.20 | -0.14 |
| Case 2b | Chinese | -0.20 | -0.14 |
| Case 3a | Chinese | 1.00 | 1.00 |
| Case 3b | Chinese | -0.20 | -0.14 |
| Case 4a | Chinese | -0.20 | -0.14 |
| Case 4b | Chinese | -0.20 | -0.14 |
| Case 5a | Chinese | -0.20 | -0.14 |
| Case 5b | Chinese | -0.20 | -0.14 |
| Case 6a | English | -0.14 | -0.11 |
| Case 6b | English | -0.14 | -0.11 |
| Case 7a | English | -0.14 | -0.11 |
| Case 7b | English | -0.14 | -0.11 |
| Case 8a | English | -0.14 | -0.11 |
| Case 8b | English | -0.14 | -0.11 |
| Case 9a | English | -0.14 | -0.11 |
| Case 9b | English | -0.14 | -0.11 |
| Case 10a | English | -0.14 | -0.11 |
| Case 10b | English | -0.14 | -0.11 |
| *Notes*. Textual Fleiss’ κ assessed the subset of nine textual sentiment analysis tools; overall Fleiss’ κ assessed the complete tool set including non-text analysis tools | | | |

**Table 13** Gwet’s AC1 robustness check for cross-tool agreement

| **Analysis set** | **No. of clips** | **No. of valid tool ratings** | **Observed agreement** | **Chance agreement** | **Gwet’s AC1** |
| --- | --- | --- | --- | --- | --- |
| Textual tools, all clips | 20 | 140 | 0.31 | 0.16 | 0.18 |
| Overall tools, all clips | 20 | 180 | 0.32 | 0.16 | 0.19 |
| Chinese clips, textual tools | 10 | 60 | 0.37 | 0.15 | 0.25 |
| Chinese clips, overall tools | 10 | 80 | 0.39 | 0.14 | 0.29 |
| English clips, textual tools | 10 | 80 | 0.26 | 0.16 | 0.12 |
| English clips, overall tools | 10 | 100 | 0.25 | 0.16 | 0.10 |

*Notes.* Gwet’s AC1 was computed as a robustness check for the potential sensitivity of Fleiss’ kappa to skewed category distributions. “Textual tools” refers to the nine text-based sentiment analysis tools listed in Appendix F (GPT-4o, Deepseek-R1, Google Cloud NL API, Amazon Comprehend, BERT-GO-Emotions, BERT-Multilingual, VADER, TextBlob, HanLP). “Overall tools” refers to the textual tools plus Microsoft Azure and Amazon Rekognition. Chinese and English clips were analyzed separately to examine whether the agreement pattern differed by legal-language context. N/A outputs (from Appendix F) were omitted from the relevant calculations. Categories followed the six converted valence intervals reported in Appendix F.
